# Predicting water consumption habits for seven arsenic-safe water options in Bangladesh

**DOI:** 10.1186/1471-2458-13-417

**Published:** 2013-05-01

**Authors:** Jennifer Inauen, Robert Tobias, Hans-Joachim Mosler

**Affiliations:** 1 Environmental and Health Psychology, Department of Environmental Social Sciences, Eawag: Swiss Federal Institute of Aquatic Science & Technology, Überlandstrasse 133, Dübendorf, 8600, Switzerland; 2 Developmental and Health Psychology, Department of Psychology, University of Konstanz, P.O. Box 5560, Konstanz, 78457, Germany

**Keywords:** Habitual health behavior, Social-cognitive predictors, Arsenic-safe drinking water, Regression, Bangladesh

## Abstract

**Background:**

In Bangladesh, 20 million people are at the risk of developing arsenicosis because of excessive arsenic intake. Despite increased awareness, many of the implemented arsenic-safe water options are not being sufficiently used by the population. This study investigated the role of social-cognitive factors in explaining the habitual use of arsenic-safe water options.

**Methods:**

Eight hundred seventy-two randomly selected households in six arsenic-affected districts of rural Bangladesh, which had access to an arsenic-safe water option, were interviewed using structured face-to-face interviews in November 2009. Habitual use of arsenic-safe water options, severity, vulnerability, affective and instrumental attitudes, injunctive and descriptive norms, self-efficacy, and coping planning were measured. The data were analyzed using multiple linear regressions.

**Results:**

Linear regression revealed that self-efficacy (*B* = 0.42, *SE* = .03, *p* < .001), the instrumental attitude towards the safe water option (*B* = 0.24, *SE* = .04, *p* < .001), the affective attitude towards contaminated tube wells (*B* = −0.04, *SE* = .02, *p* = .024), vulnerability (*B* = −0.20, *SE* = .02, *p* < .001), as well as injunctive (*B* = 0.08, *SE* = 0.04, *p* = .049) and descriptive norms (*B* = 0.34, *SE* = .03, *p* < .001) primarily explained the habitual use of arsenic-safe water options (*R*^*2*^ = 0.688). This model proved highly generalizable to all seven arsenic-safe water options investigated, even though habitual use of single options were predicted on the basis of parameters estimated without these options.

**Conclusions:**

This general model for the habitual use of arsenic-safe water options may prove useful to predict other water consumption habits. Behavior-change interventions are derived from the model to promote the habitual use of arsenic-safe water options.

## Background

The lack of safe drinking water, particularly in developing countries, is becoming an increasingly serious global topic. Worldwide, approximately one billion people do not have access to safe water [1]. One source of contamination is the naturally occurring arsenic in groundwater, which affects millions of people in many countries worldwide [2]. Continuous arsenic consumption can lead to arsenicosis, which includes skin diseases, various cancers, and other chronic diseases that may result in death [3]. Preventing arsenic-related illnesses by providing arsenic-safe water is vital because many of the health effects are irreversible.

In Bangladesh, where 20 million people are at the risk of drinking water from arsenic-contaminated wells, the following eight low-cost safe water alternatives are being implemented to provide arsenic-safe and pathogen-free water: (a) deep tube wells that tap deeper, arsenic-free aquifers, (b) rainwater harvesting, (c) household arsenic removal filters, (d) community arsenic removal filters, (e) rural piped water supply that provide safe water by distributing deep tube well or filtered pond and river water, (f) pond sand filters, which remove pathogens from arsenic-free surface water, (g) dug wells, that is, arsenic-safe, very shallow hand dug wells, and (h) well-switching, that is, switching to neighbors’ uncontaminated shallow tube wells. However, people often do not use or maintain the use of available safe water alternatives [4,5], which has been attributed to people’s socioeconomic characteristics and low risk awareness [6,7]. However, increased awareness often does not translate into increased risk mitigation behavior [8]. Psychological research suggests that health behaviors (e.g., dietary behaviors, smoking cessation, and exercise behaviors) are influenced by social-cognitive factors, such as attitudes, norms, and self-regulatory processes [9-11]. Extant studies show that social-cognitive factors are predictive of safe water consumption [12-14]. For arsenic, to our knowledge, only one study has investigated the social-cognitive predictors based on health-psychological theory; this study found that the quantity of deep tube well water consumed depends primarily on the descriptive norm [14], that is, the perceptions about which behaviors are typically performed [15]. Further behavior-influencing factors that were identified included self-efficacy (belief in one’s capabilities to organize and execute the courses of action required to manage prospective situations [16]), the injunctive norm (perceptions regarding which behaviors are typically approved or disapproved [17]), and preference for the taste of the water from the arsenic-contaminated tube well. However, several variables in the model (e.g., self-efficacy) were imprecisely defined and conceptualized, which may have led to interdependencies between predictors. More importantly, some potentially influential social-cognitive factors, such as coping planning, were not included in the model. Building a more comprehensive model, which can be generalized to more safe water options, would be beneficial to provide a starting point for developing behavior-change interventions [18].

### Social-cognitive predictors of safe water consumption

A helpful framework of potentially influential factors impacting the use of safe drinking water options is the Health Action Process Approach (HAPA; [19]). This theory has been successfully employed for explaining many health behaviors [19]. The HAPA specifies risk perception, outcome expectancies, self-efficacy, and planning as behavior-influencing factors. However, to gain more intervention-relevant information (for interventions corresponding to behavior-influencing factors, see [18]), it has been suggested that risk perception and outcome expectancies be divided into several factors, as displayed in the RANAS Model (Risk, Attitude, Norms, Ability, and Self-Regulation) [18]. Risk perception can be divided into perceived vulnerability, that is, a person’s subjective perception of his/her risk of contracting a particular condition or illness, and perceived severity, that is, a person’s perception concerning the seriousness of the consequences of contracting a particular condition or illness [20,21]. It has been proposed that outcome expectancies be divided into social, physical, and emotional components [19]. Social influences can be differentiated into the aforementioned injunctive and descriptive norms. Corresponding to the physical and emotional components of the HAPA [19], affective (e.g., enjoyable/not enjoyable) and instrumental (e.g., beneficial/harmful) evaluations of the behavior can be distinguished [22].

As specified above, two other components of the HAPA [22] are self-efficacy and planning. The planning component of the HAPA [22] comprises action and coping planning. Coping planning is the presumption of possible barriers and the invention of ways to overcome them [22]. Action planning is the formation of plans to initiate a new behavior, which is important for adoption but not for habitual behavior; therefore, action planning will not be considered in our study.

Besides the psychological factors related to the target behavior, we argue that it is important to consider the social-cognitive factors for regarding the competing behavior. Every behavior has an alternative, and new behaviors are weighed against the old behaviors that serve similar purposes. For the case of drinking water in Bangladesh, we have to consider not only the factors that support the use of arsenic-safe water options but also the ones that favor the use of arsenic-contaminated wells.

### Towards understanding, predicting, and creating habitual behaviors

To avoid most health threats, including arsenicosis, it is necessary to not only adopt a healthy behavior but also to maintain this practice over time. Therefore, the goal of every behavior change campaign is to induce long-lasting behavior change. One indicator of sustained behavior is habit. Habits facilitate intended behaviors because they require reduced cognitive effort [23]. Therefore, as also suggested by Verplanken and Wood [24], we propose that developing habits should be considered an important additional goal of behavioral change campaigns. Consequently, a habit should be considered a dependent variable in statistical investigations to enable the identification of its determinants, which can be targeted in behavioral change campaigns cf. [25,26]. However, explaining and predicting habits alone does not seem sufficient. In the case of promoting new behaviors, some individuals may already be exhibiting these new behaviors, but they may not have developed into habits yet. However, other individuals may not be exhibiting the behavior yet. To consider all individuals simultaneously, the actual behavior and the stability of this behavior should be considered (i.e., the habit). In this study, we use the concept of *habitual behavior*, which considers both the habit and the actual behavior.

### The present study

In summary, this study aimed to develop a simple linear model to explain and predict water consumption habits based on the consumption data of arsenic-safe water in Bangladesh. The model to be developed in this study aims to predict an entire class of specific behaviors, that is, the habitual use of all arsenic-safe water options. Therefore, the generalizability of this model becomes an issue. We must investigate the validity of this model in predicting the habitual use of each specific arsenic-safe water option that it is presumed to explain. In conclusion, this study investigates the following two research questions: (1) Which factors are related to the habitual use of arsenic-safe water options and what are their parameter estimates? (2) How effectively does this general model predict the use of specific water options based on the model for all water options for which it was developed (i.e., all arsenic-safe water options in Bangladesh)?

To investigate our first research question, the following hypotheses were derived from the HAPA [19]: Increased habitual use of arsenic-safe water options will be in line with increased perceived severity (H1), increased perceived vulnerability (H2), more positive affective (H3) and instrumental attitudes (H4) toward the arsenic-safe water option, more favorable injunctive (H5) and descriptive norms (H6) towards the arsenic-safe option, increased self-efficacy (H7), and more detailed coping planning (H8). Therefore, we expect less habitual use of arsenic-safe water options to be associated with increased affective attitudes (H9) and increased descriptive norms (H10) regarding arsenic contaminated tube wells.

For the second research question, separate forecasts for the habitual use of each arsenic-safe water option are derived from the model using parameter values that were estimated for the entire sample. The findings of this study will contribute to improving the understanding and prediction of the habitual safe water consumption. Furthermore, theory-based behavior-change interventions to enhance the habitual use of arsenic-safe drinking water options are derived from the results of this study.

## Methods

### Design and setting

A cross-sectional household survey was conducted in rural Bangladesh during November 2009. The study areas comprised 40 villages in six arsenic-affected districts of Bangladesh, which were purposefully selected due to the presence of high arsenic-contamination and the availability of one or more of the seven arsenic-safe water options considered in this study, which are specified in the next subsection. The following six districts were included in this study and the available arsenic-safe water options in these districts are indicated in parentheses: Satkhira (community arsenic removal filters), Khulna (dug wells and well-switching), Bagerhat (pond sand filters), Comilla (piped water supply, community arsenic removal, dug wells, well-switching, rainwater harvesting, and household arsenic removal filters), Munshiganj (well-switching and rainwater harvesting), and Brahmanbaria (piped water supply). Note that this paper focuses on all arsenic-safe water options in general, whereas the seven surveyed options in particular are published elsewhere [27].

### Participants and procedures

The criteria for study participation were as follows: (a) exposure to the risk of drinking arsenic-contaminated water (i.e., households who reportedly owned or had access to an arsenic-contaminated tube well, in case they did not own a well), and (b) access to one of the following seven arsenic-safe water options at the time of the survey: household arsenic removal filters, household rainwater harvesting, community arsenic removal filters, rural piped water supply, pond sand filters, dug wells, and well-switching. Note that criterion (b) does not presuppose that people were using the available safe water options at the time of the survey. We excluded the people with access to deep tube wells from the study, because we had previously studied the use of deep tube wells [14]. Based on sample size estimation with GPOWER [28], the aim was to survey 125 households for each water option to detect small to medium differences with a Type I error probability of 0.05 and a statistical power of 0.95 when comparing two single options.

The survey was conducted by 12 professional Bangladeshi interviewers. Conducting structured psychological surveys in rural areas of developing countries is always a challenge. Most people in developing countries, particularly in rural areas, are not used to answering psychological questions and the scaled response format was a novel approach for them. Therefore, we devoted considerable time and effort to training interviewers and rehearsing interviewing techniques with them for facilitating respondents’ understanding of the survey and enabling them to answer questions appropriately. Two local supervisors assisted the interviewers and performed data quality checks with the first author and a master’s student. The interviewers selected households within a given study area by random-route sampling [29]. This method entailed the team of interviewers to first meet at a central point in the village and then spread into different directions. On their routes, interviewers selected every third household and screened whether these households met the inclusion criteria. Subsequently, the interviewers enquired which household member was responsible for water provision in each of these households. After obtaining their informed consent, the interviewers conducted the interviews with the household members responsible for water provision. Interview durations ranged from 1 to 1.5 hours.

A total of 872 households were interviewed: 126 households owned a household arsenic-removal filter, 123 households owned or had access to a rainwater harvester, 124 households had access to dug wells, 124 households had access to pond sand filters, and 125 households each had access to piped water supply, community arsenic removal filters, or a neighbor’s uncontaminated tube well (i.e., well-switching). Although interviewers emphasized that participation was voluntary, none of the approached households refused the interview. However, 30% of the potential participants who had once received household filters stated that they had never received these, which could be interpreted as a refusal.

### Measures

A structured questionnaire was specifically developed for this study (see Additional file 1). The majority of the items were derived from extant literature ([14], if not indicated otherwise) and adapted for Bangladesh and the water consumption context wherever necessary.

The questionnaire included structured items addressing water consumption behavior, psychological variables, and sociodemographic information, as well as open questions dealing with the advantages and disadvantages of different arsenic-safe water options. The questionnaire was translated into Bengali and re-translated into English to verify the quality of the translation. During questionnaire preparation and pretest, we worked closely with local collaborators to ensure that the rural population understood our question and answer formats.

The survey items were averaged to build scales. Unipolar items offered five response options (from 0 to 4), whereas bipolar items offered the following 9-point scales: 4 points in one direction (e.g., from “rather dislike it” to “dislike it very much”), 4 points in the opposite direction (e.g., from “rather like it” to “like it very much”), and one neutral point (e.g., “neither particularly like it nor dislike it”).

#### Outcome variable: habitual behavior

As proposed in the introduction, we employed a new composite measure for habitual behavior. It comprised a measure for current behavior and the following three components of self-reported habit: perceived habit, automaticity, and regularity. Such a scale is a continuum that permits the simultaneous consideration of current users and non-users of a target behavior, including their degrees of habit to perform it. One side of this continuum represents the people who are not performing the behavior currently and have never done so in the past (i.e., zero habit). The other side of the continuum represents people who are currently performing the behavior with great automaticity and regularity (i.e., great habit strength). In between these extremes are people who are not currently performing the behavior with regularity. These are, for example, people with some habit for the behavior, but who do not currently perform it (e.g., because they just moved to a new area). Alternatively, these may be people who have just started performing the behavior, but this behavior has not yet developed into a habit; therefore, their practices are vulnerable to change.

The habitual behavior scale was built by averaging the proportion of arsenic-safe water of the total drinking water consumption, the automaticity and regularity components of the Self-Report Habit Index (SRHI; [30]), and perceived habit. Please see Additional file 2: Table S1 in the supporting information for details.

#### Psychological predictors of habitual behavior

The supporting information (Additional file 2: Table S1) provides a detailed overview of the conceptualization of the severity, vulnerability, affective attitudes, instrumental attitudes, injunctive and descriptive norms, self-efficacy, coping planning, and their operationalization.

### Data analysis

Simultaneous multiple linear regressions were computed to explain the habitual use of arsenic-safe water options by the psychological predictors. Assumptions of linearity, homoscedasticity, and normally distributed error terms were met for the entire sample and all the sub-samples. Multicollinearity was a minor issue for the majority of the variables; most variance inflation factors (VIF) were smaller than 1.8. However, some multicollinearity was found for the injunctive norm and self-efficacy (VIF_max_ = 2.8). The intercorrelations confirmed these observations. Furthermore, they reveal particular interrelatedness of the affective attitude toward arsenic-safe water options, the injunctive norm, and self-efficacy, as well as of self-efficacy and coping planning.

The model developed in this study claims not only to explain the habitual use of one specific water option but also to be generalizable to an entire class of arsenic-safe water options. Therefore, the model cannot be evaluated merely by fitting it to the data; the generalizability of its data-fitting abilities [31] must be tested. The method of choice for such a task is cross validation, where model parameters (here, the parameters b_0_ to b_p_ of the regression equation Y = b_0_ + b_1_*X_1_ + … + b_p_*X_p_) are initially estimated with one sub-set of the data and subsequently with another sub-set, and the habitual behavior is predicted with the previously estimated parameters. If the two sub-sets of data refer to the habitual use of different arsenic-safe water options, this test not only provides information about the generalizability of the model to other samples but also to the habitual use of other water options.

Overall, the model was fitted to nine data sets (i.e., sub-samples of the total sample). In Estimate 1, the entire sample was used to estimate the regression parameters. With these parameter estimates, the forecasts of the habitual use of the seven arsenic-safe water options were computed for each participant. Initially, the parameter estimates from the regression were entered into the regression equation and the habitual behavior of each participant was predicted by the model (Estimate 1). To obtain a measure of how effectively the model predicted the habitual use of each arsenic-safe water option, we calculated the Pearson correlation between the predicted habitual behavior value and the self-reported habitual behavior of each participant. This correlation was computed for each of the seven arsenic-safe water options and for the total sample. The squared values of these correlations (i.e., *R*^*2*^) provides the first indication regarding the generalizability of the model for each of the seven arsenic-safe water options: the smaller the variation of the model fit for a specific water option compared to the fit to the entire sample (i.e., all water options), the higher the generalizability of this particular option. Therefore, it is possible to identify the habitual use of which water options can be forecasted effectively. In further generalizability tests, the procedure explained above was repeated for other sub-sets of the data. Seven estimates were calculated by employing the data of six water options and omitting the data of one water option (Estimates 2 to 8). This revealed how effectively the model would perform if it was applied to a similar water option that was not included in the data set for estimating the parameters. Once again, this investigation can be used to identify the habitual use of the water options that are difficult to forecast using this model. Finally, two more strict cross-validations were calculated. For Estimate 9, the two best-explained water options were excluded from the regression parameter estimation, whereas for Estimate 10, the two worst-explained water options were excluded for this estimation. Subsequently, these parameters were entered into the regression equation to forecast the habitual use of each of the water options once with parameters from Estimate 9 and once with those from Estimate 10. Splitting the sample in this manner maximized the differences among the subsamples, and thus maximized the forecasting difficulties. All analyses were calculated using SPSS 18.0.

### Ethics statement

This study was conducted in strict compliance with the ethical principles of the American Psychological Association (APA) and the Declaration of Helsinki. It underlies the ethics review board of the ETH, Swiss Federal Institute of Technology Zurich. This review board exempts the survey studies that do not comprise an intervention from obtaining an ethical approval: http://www.vpf.ethz.ch/about/commissions/EK.

## Results

### Descriptive statistics

The socioeconomic characteristics of the households and the person responsible for the collection of drinking water for each of the households can be found in Table 1. Overall, 65.8% (574) of the households used the available arsenic-safe water options for consumption. However, user rates considerably differed among the various water options. At the time of the interview, of the households to whom the respective water option was available, 36.6% (45) used rainwater harvesters, 92.9% (117) used household filters, 73.6% (92) used community filters, 51.6% (64) used pond sand filters, 85.6% (107) used piped water supply, 48.4% (60) used dug wells, and 72.2% (89) used neighboring arsenic-safe wells. On average, habitual behavior was medium (*M* = 2.2, *SD* = 1.4, *N* = 872); however, this differed among the safe water options. Habitual behavior averaged 1.1 (*SD* = 0.6) for households with rainwater harvesters, 3.1 (*SD* = 0.9) for those with household filters, 2.58 (*SD* = 1.3) for households with access to community filters, 1.7 (*SD* = 1.7) for people with access to pond sand filters, 3.0 (*SD* = 0.9) for those with access to piped water supply, 1.6 (*SD* = 1.5) for households with access to dug wells, and 2.4 (*SD* = 1.2) for those with access to neighboring arsenic-safe wells. Further descriptive statistics and intercorrelations of habitual behavior and all psychological predictors in the study can be found in Table 2.

**Table 1:** **Socioeconomic characteristics of respondents/households (*N*** **= 872)**

**Characteristics**	***n***	***%***
Gender (% female)	618	70.9
Literacy rate	552	64.6
Religion (% Muslim*)	782	89.7
Occupation of participant:		
Housewife	582	66.7
Independent work	94	10.8
Agriculture	90	10.3
Other	106	12.2
Occupation of household head:		
Independent work	352	40.4
Agriculture	255	29.2
Formal employment	105	12.0
Other	164	18.8
	*M*	*SD*
Age	37.6	12.6
Education (years)	4.8	4.2
No. of people living in household	5.4	2.3
Monthly income (BDT)	8961	11649

*Note. BDT*, Bangladeshi Taka (77 BDT was app. 1 US Dollar), *all other respondents reported Hinduism as their religion.

**Table 2:** **Descriptive statistics and correlations for outcome and independent variables (*N*** **= 872)**

		***M***	***SD***	**Skew**	**Pearson correlations**^**a**^									
**Variable**		**(Q1)**	**(Q2)**	**(Q3)**	**1**	**2**	**3**	**4**	**5**	**6**	**7**	**8**	**9**	**10**
1.	Habitual behavior	(0.75)	(2.88)	(3.38)										
2.	Severity	3.33	0.71	−0.87	**0.20**									
3.	Vulnerability	−1.34	2.19	0.42	**−0.59**	**−0.14**								
4.	Affective attitude arsenic-safe option	2.51	1.47	−1.83	**0.50**	**0.21**	**−0.30**							
5.	Instrumental attitude arsenic-safe option	2.38	0.76	−0.22	**0.29**	**−0.22**	**−0.10**^**b**^	**0.24**						
6.	Affective attitude contaminated tube well	−1.42	1.88	0.57	**−0.32**	**−0.27**	**0.29**	**−0.36**	−0.04^f^					
7.	Injunctive norm arsenic-safe option	2.57	1.08	−0.76	**0.52**	**0.46**	**−0.38**	**0.62**	**0.08**^**c**^	**−0.49**				
8.	Descriptive norm arsenic-safe option	1.50	0.93	0.38	**0.49**	**0.12**	**−0.27**	**0.27**	**0.12**^**d**^	**−0.13**	**0.28**			
9.	Descriptive norm contaminated tube well	1.22	0.75	1.05	**−0.31**	**−0.07**^**e**^	**0.32**	**−0.17**	**−0.11**^**d**^	**0.21**	**−0.23**	**−0.29**		
10.	Self-efficacy arsenic-safe option	2.73	1.23	−0.71	**0.67**	**0.21**	**−0.52**	**0.52**	**0.23**	**−0.38**	**0.55**	**0.43**	**−0.32**	
11.	Coping planning	1.76	1.11	0.20	**0.37**	**−0.16**	**−0.29**	**0.31**	**0.22**	**−0.17**	**0.31**	**0.31**	**−0.21**	**0.52**

All variables ranged from 0 to 4, except for vulnerability, the affective attitudes and the injunctive norm, which ranged from −4 to 4. In parentheses: Quartiles (Q) are displayed due to the non-normal distribution of habitual behavior; Q1 = 25%, Q2 = 50%, Q3 = 75%. ^a^Except for correlations with habitual behavior. These are Spearmen correlations due to the non-normality of habitual behavior. Boldface: significant with *p* < .001, except for the following: ^b^*p* = .003; ^c^*p* = .015; ^d^*p* = .001; ^e^*p* = .046. Not significant: ^f^*p* = .248.

### Overall model for explaining the habitual use of arsenic-safe water options

All psychological variables were significantly correlated with habitual behavior (see Table 2). The final regression model was computed after eliminating five outliers with residuals greater than three. We found that six of the ten psychological predictors significantly contributed to explaining the habitual use of arsenic-safe water options (see Table 3). In line with our hypotheses, the instrumental attitudes toward arsenic-safe water options (H4), injunctive norm towards arsenic-safe water options (H5), descriptive norm towards arsenic-safe water options (H6), self-efficacy (H7), and affective attitude towards the contaminated tube well (H9) significantly predicted the habitual use of arsenic-safe water options. The strongest predictors of habitual behavior were self-efficacy, the descriptive norm towards the arsenic-safe water option, and the instrumental attitude towards the arsenic-safe water option. Furthermore, vulnerability was significantly associated with habitual use of arsenic-safe water options. However, contrary to our hypothesis, vulnerability (H2) was negatively associated with habitual behavior. Finally, severity (H1), the affective attitude towards the safe water option (H3), the descriptive norm towards contaminated or untested tube well (H8), and coping planning (H10) were not significantly associated with habitual use of arsenic-safe water options. The overall model effectively explained the habitual use of arsenic-safe water options (*R*^*2*^ = 0.688).

**Table 3:** **Simultaneous multiple linear regression of the habitual use of an arsenic-safe drinking water option (*n*** **= 867)**

				**95% CI for B**	
**Predictors**	***B***	***SE B***	***p***	***LL***	***UL***
(Constant)	−0.54	0.20	0.006	−0.92	−0.15
Severity	0.00	0.05	0.971	−0.09	0.09
Vulnerability	−0.20	0.02	0.000	−0.23	−0.17
Affective attitude arsenic-safe option	0.00	0.03	0.908	−0.05	0.05
Instrumental attitude arsenic-safe option	0.24	0.04	0.000	0.16	0.31
Affective attitude contaminated tube well	−0.04	0.02	0.024	−0.07	−0.01
Injunctive norm arsenic-safe option	0.08	0.04	0.049	0.00	0.15
Descriptive norm arsenic-safe option	0.34	0.03	0.000	0.27	0.40
Descriptive norm contaminated tube well	−0.02	0.04	0.588	−0.10	0.06
Self-efficacy arsenic-safe option	0.42	0.03	0.000	0.36	0.49
Coping planning	0.03	0.03	0.390	−0.03	0.09

*Note.* CI, Confidence interval; *LL*, Lower limit; *UL*, Upper limit. *SE B*, Standard error of unstandardized regression parameter B.

Habitual behavior and predictors ranged from 0 to 4, except for vulnerability, the affective attitudes and the injunctive norm, which ranged from −4 to 4.

Standardized parameters (β) are not displayed due to the non-normal distribution of the outcome variable. *R*^*2*^ = 0.688, *F*(10, 866) = 188.41, *p* < .001.

### Generalizability of the model for different arsenic-safe water options

The results regarding the generalizability of the model are displayed in Table 4. Furthermore, the regression parameters for each estimate can be found in the Additional file 2 (pp. S-5 to S-6). The regression parameters that were estimated with the total sample (Table 3) effectively predicted the habitual use for each of the seven arsenic-safe water options (see Table 4, Estimate 1). In particular, the habitual use of pond sand filters and community arsenic removal were effectively predicted, with explained variances of 0.801 and 0.748, respectively. Moreover, habitual use of well-switching, dug wells, and piped water supply achieved high predictive values (*R*^*2*^ ranged from 0.640 to 0.646). The lowest explained variances were found for household arsenic removal (*R*^*2*^ = 0.510) and rainwater harvesting (*R*^*2*^ = 0.539). This indicates that it is more difficult to forecast the habitual use of these safe water options using this model than the other behaviors.

**Table 4:** **Explained variances (*R***^***2***^**, all*p*** **< .001) of predictions of habitual use for different arsenic-safe water options by parameters estimated with sub-samples of the total sample**

	**1**	**2**	**3**	**4**	**5**	**6**	**7**	
**Estimate (sub-samples)**	**Rainwater harvesting**	**Household arsenic removal**	**Commun. arsenic removal**	**Pond sand filter**	**Piped water supply**	**Dug well**	**Well-switching**	**All**
Estimate 1 (total sample)	0.539	0.510	0.748	0.801	0.640	0.646	0.643	0.688
Estimate 2 (without 1)	**0.524**	0.539	0.763	0.806	0.647	0.657	0.631	0.681
Estimate 3 (without 2)	0.539	**0.448**	0.743	0.800	0.627	0.635	0.645	0.687
Estimate 4 (without 3)	0.545	0.520	**0.735**	0.793	0.634	0.640	0.646	0.689
Estimate 5 (without 4)	0.546	0.522	0.732	**0.789**	0.637	0.646	0.635	0.689
Estimate 6 (without 5)	0.540	0.494	0.743	0.798	**0.621**	0.646	0.638	0.689
Estimate 7 (without 6)	0.535	0.485	0.743	0.800	0.640	**0.625**	0.650	0.688
Estimate 8 (without 7)	0.536	0.524	0.755	0.804	0.657	0.650	**0.637**	0.689
Estimate 9 (without 3 and 4)	0.557	0.530	**0.704**	**0.771**	0.620	0.634	0.635	0.683
Estimate 10 (without 1 and 2)	**0.525**	**0.499**	0.767	0.808	0.638	0.653	0.634	0.679
*n* (per predicted option)	125	126	122	122	125	122	125	867

*Note.* Explained variance of predictions for options from estimates that were calibrated without these options are in boldface.

A first test of the generalizability of the overall model was to predict the habitual use of each safe water option based on the regression parameters estimated without the data from the particular option (Estimates 2 to 7). The results of these tests demonstrated high robustness for the majority of the predictions. The explained variances were almost the same for most of the options. The largest change in explained variance was found for the habitual use of household filters, which dropped from 0.510 to 0.448.

In a second and stricter cross validation, we excluded the best-explained options from the parameter estimation (Estimate 9). The results of this test revealed a surprising generalizability of the model. The drops in explained variance compared to the reference estimates were minimal, and more than half of the variance was explained for all behaviors, except for household arsenic removal.

## Discussion

This study investigated the importance of psychological factors derived from health behavior theories to predict the habitual use of arsenic-safe drinking water options. Furthermore, we investigated how effectively this general model can predict the use of specific arsenic-safe water options. Results from a large household survey in Bangladesh showed that the habitual use of arsenic-safe water options is strongly associated with self-efficacy as well as the descriptive norm and instrumental attitude towards the arsenic-safe water option. Moreover, these related factors were identified: the injunctive norm, vulnerability, and attitude towards the health-risking water option (i.e., arsenic-contaminated or untested shallow tube wells). This corroborates recent findings that social-cognitive factors are highly predictive of safe water consumption [12,13]. The model proved highly generalizable to explain the habitual use of all seven arsenic-safe water options investigated in this study, even when the habitual use of these options was predicted based on parameters estimated without the data of these options.

The results of this study support the findings of Mosler et al. [14] that norms and self-efficacy are major drivers of safe water collection. These results can be regarded as quite generalizable, because they were replicated in the present study for various safe water options, different operationalization of constructs, and greater sample size.

Regarding the psychological predictors of the habitual use of arsenic-safe water options, contrary to our hypotheses, we found no significant associations between habitual behavior and severity, affective attitude towards the safe water option, descriptive norm regarding the contaminated or untested shallow tube well, and coping planning. However, discarding these factors as unimportant may be premature, because our data also revealed some interrelatedness between the constructs. For example, there was an association between the injunctive norm and affective attitude towards the arsenic-safe water option. From a modeling perspective, this indicates that it may be beneficial to combine attitudinal and normative expectancies into one factor, outcome expectancies, as proposed by the social cognitive theory [32] and the HAPA [19]. From the perspective of intervention design, this result suggests that these factors should not be entirely neglected when designing campaigns aimed at increasing arsenic-safe water consumption.

As a separate influence, our study demonstrated that it might be valuable to consider the behavior alternative independently of the target behavior: the affective attitude towards the contaminated or untested tube well was negatively associated with habitual behavior.

One surprising result of our study is the negative association between vulnerability and habitual behavior. Although initially this result may seem to be in contrast with theory, which assumes more health-protective action for people with higher vulnerability [33], one possible interpretation may be that people who engage in health-protective actions consequently feel less vulnerable to health threats. Longitudinal research may establish the causality of this relationship more conclusively. Moreover, questions assessing vulnerability should include a reference to the behavior to avoid confusions of causality.

One strength of this study is the simultaneous investigation of several behaviors (i.e., several safe water options), which permits the development and test of a general model for an entire class of specific behaviors. In numerous analyses, the general model not only proved successful for forecasting the habitual use of each of the specific water options investigated but also proved apt for forecasting the habitual use of water options with model parameters that were estimated without these options. However, the habitual use of some water options was found to be more difficult to forecast than other options. The habitual use of rainwater harvesting and household filters was not explained as effectively as that for the other options. One reason for this may be that both of these are household-based options, whereas the other arsenic-safe water options are designed for communities or groups of households. This explanation is supported by the fact that these options were better forecasted when community arsenic removal and pond sand filters, the two best-explained options that are both clear-cut community options, were excluded from the estimation. Since any model testing can only be conducted with available data, it remains unknown whether the good generalizability of the model, as observed in this study, holds for water options not considered in this study. Therefore, in future studies, the generalizability of the model to other populations (e.g., in other countries) and water consumption habits that were not included in this study (e.g., chlorination, boiling) should be investigated.

One of the limitations of this study is its cross-sectional design. The causality of the relationships cannot be investigated with such data. Longitudinal studies with controlled manipulations of the parameters are necessary for investigating whether the relationships discovered by us are of causal nature and if so, for determining the direction in which this causality runs. Such data will soon become available because behavior-change campaigns for enhancing the habitual use of arsenic-safe water options are in preparation. From a behavior change perspective, another shortcoming of this study is the inclusion of the very general concept of self-efficacy in the model. In contrast to the other constructs employed, it is difficult to target self-efficacy directly by commonly used interventions owing to its broadness. Future studies should therefore distinguish between different types of self-efficacy (e.g., maintenance and recovery self-efficacy, [19]). Furthermore, determinants of self-efficacy, such as those suggested by social learning theory (e.g., verbal persuasion, [34]), should be explored. Moreover, the measures for habitual behavior used in our study were self-report and therefore potentially prone to reporting bias. However, we argue that a potential bias may have been reduced by the combination of current behavior and perceived habit. Questions regarding water consumption are arguably more vulnerable to social desirability, because the desired answer can be easily guessed. However, questions on perceived habit are more abstract. Therefore, the purpose of these questions and their socially “correct” answers may not be as apparent as those for water consumption. If future investigations can find support for this assumption, then this would be another advantage of employing this behavioral measure, even in related fields. Future investigations of habitual behavior may also explore whether ascribing stronger weightage to current behavior in the habitual behavior scale may be beneficial.

A general model for predicting water consumption habits is highly valuable for planning, guiding, and evaluating campaigns aimed at encouraging these habits. The model factors can be systematically targeted in behavior-change campaigns. Our results suggest a combination of self-efficacy and normative interventions. One intervention strategy that targets both these factors is modeling (i.e., observational learning; [34]). Motivated members of the target communities who are already using arsenic-safe water options can be recruited as promoters. During their visits, the promoters would assist households to locate arsenic-safe water options in their neighborhoods. Subsequently, the promoters and the people responsible for water collection in their respective households would go together to collect water from this identified option. This will enable the target people to understand that the water source is indeed accessible, which should increase self-efficacy and lower the perceived expenditure of time (i.e., increase instrumental attitudes). Simultaneously, the descriptive norm becomes more salient when promoters meet community members who are using arsenic-safe water. Moreover, the injunctive norm may increase when promoters talk favorably regarding the use of arsenic-safe water options. Therefore, it is ideal to recruit opinion leaders as promoters (i.e., people whose opinion is valued by most community members). Finally, to increase perceived vulnerability, it is advisable that promoters explain the concepts of arsenic, arsenicosis, and arsenic-safe water options to community members by, for example, demonstrating pictograms.

## Conclusions

This study yielded strong support for the assumption that social-cognitive factors, particularly self-efficacy and the descriptive norm, are strongly predictive of safe water consumption. Owing to its generalizability, the model can be employed for predicting and serving as a basis for promoting the habitual use of any arsenic-safe water option. Furthermore, it may even be useful for understanding and promoting behaviors that were not considered in the estimation of the parameters. This is of particular importance because an increasing number of technical solutions for providing safe drinking water are being developed. Thus, the model presented can be used to develop theory-based interventions targeting the determinants of habitual behavior.

## Competing interests

The authors declare that they have no competing interests.

## Authors’ contributions

JI designed the study, drafted the questionnaire, collected the data, performed the analyses, and drafted the manuscript. RT participated in designing the study and in drafting the questionnaire, supported the data analysis, and contributed to drafting the manuscript. HJM participated in designing the study and to drafting the manuscript. All authors read and approved the final manuscript.

## Pre-publication history

The pre-publication history for this paper can be accessed here:

http://www.biomedcentral.com/1471-2458/13/417/prepub

## Supplementary Material

Additional file 1 **Questionnaire.** The file contains the English translation of the original survey questionnaire. Click here for file

Additional file 2 **Supporting information.** The file contains a table with detailed definitions of all psychological constructs investigated, including their operationalization and internal consistency measures. Furthermore, the parameter estimates and their confidence intervals from all regression models are displayed and discussed. Click here for file
