# Supplementary material for: Predicting water consumption habits for seven arsenic-safe water options in Bangladesh
Source: BMC Public Health. 2013 May 1;13:417. doi: 10.1186/1471-2458-13-417 (PMC3649919; doi:10.1186/1471-2458-13-417)
Supplement: Additional file 1 — Questionnaire. The file contains the English translation of the original survey questionnaire. [file 1471-2458-13-417-S1.pdf]

Social acceptance and use of arsenic mitigation options in Bangladesh

Pilot interviews November/December 2009
Questionnaire

For the interviewers: Please write down **which** households (their **assigned household number**) did **not** want to be interviewed **before** you found this household who takes part in the interview.

....

Please interview the person of the household that is responsible for the water for the family!

Introduction

Please introduce yourself!

Hello, my name is ………………………….. and I am working for Eawag, the Swiss Federal Institute of Aquatic Science and Technology. We are conducting a research study on household water consumption. If you don't mind, I would like to interview you about your water consumption preferences. It will take about 45 minutes. Do you have the time for the interview? We are also interviewing other households in your community as well as other communities in Bangladesh. The results will be treated anonymously. We are not interested in any particular answers, just in the answers that really represent your opinion. We would like to know why people are doing what they are doing so that we can improve the drinking water situation depending on this information. It helps us most if you answer as honest and properly as possible. Please help us in finding out how things really are!

General information regarding the interview

Start time: ....

B101 ID number: ....

B102 Date of the interview: ....

B103 Number & name of the interviewer: ....

B104 District: 1 ❑ Satkhira 2 ❑ Munshiganj 3 ❑ Comilla 4 ❑ Other:..

B105 Upazila: 1 ❑ Assassuni 2 ❑ Sirajdikhan 3 ❑ Homna 4 ❑ Other:..

B106 Union: ....

B107 Ward: ....

B108 Village: ....

Data of the interviewed person

B114 Name *(if they refuse, no problem)*:..

B115 Gender: 0 ❑ Male 1 ❑ Female

B116 Age: ....

B117 Respondent's occupation:
- 1 ❑ Unemployed
- 2 ❑ Housewife
- 3 ❑ Agriculture
- 4 ❑ Informal employment
- 5 ❑ Formal employment
- 6 ❑ Independent work
- 7 ❑ Studies
- 8 ❑ Retired
- 9 ❑ Other:..

B416 Head of household's occupation:
1 ❑ Unemployed
2 ❑ Housewife
3 ❑ Agriculture
4 ❑ Informal employment
5 ❑ Formal employment
6 ❑ Independent work
7 ❑ Studies
8 ❑ Retired
9 ❑ Other:..

B118 Are you able to read or write?
1 ❑ Can neither read nor write
2 ❑ Can read only
3 ❑ Can write only
4 ❑ Can both read and write

B119 Education (years): ....

B120 Religion:
1 ❑ Muslim
2 ❑ Hindu
3 ❑ Buddhist
4 ❑ Christian
5 ❑ Sonaton
6 ❑ Other:...................................

People's problems

B147 What are the **biggest problems** you and your family are facing during everyday life? ....

....

....

Interviewer: fill in answers into spaces *Error! Reference source not found.* ***to*** *Error! Reference source not found.* ***(if not already written below).***

Which ones of the following problems are **the three most severe ones** for you? ***(Read the whole list!)***.

1 = the most severe, 2 = the 2nd most severe, 3 = the 3rd most severe

0 = it is not a problem

Problem	Severity rating
B148 Illness	
B149 Unemployment	
B150 Windstorms	
B151 Floods	
B152 Poverty	
B153 Price-hikes	
B154 Food Insecurity	
B155 Other (***fill in from above***): :..	*B155r*
B156 Other (***fill in from above***):..	*B156r*
B157 Other (***fill in from above***):..	*B157r*

B158 What are the **health threats** you and your family are most concerned about?..

....

....

Interviewer: fill in answers into spaces *Error! Reference source not found.* ***to*** *Error! Reference source not found.* ***(if not already written below).***

Which ones of the following health threats are **the three most severe ones** for you? ***(Read the whole list!)***.

1 = the most severe, 2 = the 2nd most severe, 3 = the 3rd most severe

0 = it is not a problem

Health threat	Severity rating
B159 Fever	
B160 Diarrhea	
B161 Arsenicosis	
B162 Tuberculosis	
B163 Pain	
B164 Water related illness	
B165 Other (***fill in from above***): :...	*B165r*
B166 Other (***fill in from above***): :...	*B166r*
B167 Other (***fill in from above***): :...	*B167r*

B168 What are the **water related problems** you and your family are most concerned about?.................................

....

....

Interviewer: fill in answers into spaces *Error! Reference source not found.**B173 to B175 (if not already written below).***

Which ones of the following water related problems are **the three most severe ones** for you? ***(Read the whole list!)***.

1 = the most severe, 2 = the 2nd most severe, 3 = the 3rd most severe

0 = it is not a problem

Water related problem	Severity rating
B169 Bacteria in water	
B170 Not enough water	
B171 Arsenic in water	
B172 Iron in water	
B173 Other (***fill in from above***): :...	*B174r*
B174 Other (***fill in from above***): :...	*B175r*
B175 Other (***fill in from above***): :...	*B176r*

Water consumption history

B109 Do you have a tubewell in your household? 1 ❑ yes 0 ❑ no

***If no** → B417*

B111 Has your tubewell ever been tested for arsenic? 1 ❑ yes 0 ❑ no

B112 Is your tubewell contaminated with arsenic? 1 ❑ yes 0 ❑ no

B113 Have you ever increased the depth of your tubewell? 1 ❑ yes 0 ❑ no

Water treatment use history from about 5 years ago until today:

Have you ever used any of the following household water sources directly for drinking or cooking? Are you still using it? Please tell us all the dates when you started and stopped using these water sources and please name the reasons for starting and stopping.

	**Water source type available or not?**		**Date start (MM/YY)** *_a*	**Date stop (MM/YY)** *_b*	**What was the reason why you started (e.g. promotion event etc.)?** *_c*	**Why did you stop? Please try to remember the exact cause!** *_d*
B417	Own untested tubewell (directly)	1 ❑ yes<br>0 ❑ no				
B418	Own **un**contaminated tubewell (directly)	1 ❑ yes<br>0 ❑ no				
B419	Own contaminated tubewell (directly)	1 ❑ yes<br>0 ❑ no				
B420	Own **re-sunk** tubewell (directly)	1 ❑ yes<br>0 ❑ no				
B421	Neighbor’s untested tubewell (directly)	1 ❑ yes<br>0 ❑ no				
B422	Neighbor’s **un**contaminated tubewell (directly)	1 ❑ yes<br>0 ❑ no				
B423	Neighbor’s contaminated tubewell (directly)	1 ❑ yes<br>0 ❑ no				
B424	Household filter (Sono, Alcan, Read-F)	1 ❑ yes				

B425	Household rainwater harvesting system	1 ❑ yes<br>0 ❑ no				
B426	Pond/river/canal	1 ❑ yes<br>0 ❑ no				
B427	Bottled water	1 ❑ yes<br>0 ❑ no				

If the respondent never used tubewell water for drinking or for cooking: thank respondent, ask if she or he has any questions, answer them and end interview.

Are there any of the following <u>community water sources</u> in your village? Are you using it or have you used it in the past for drinking or cooking? Please tell us all the dates when you started and stopped using these water sources and please name the reasons for starting and stopping.

	**Water treatment type**	**Available or not ?**	**Date start (MM/YY)** *_a*	**Date stop (MM/YY)** *_b*	**What was the reason you started (e.g. promotion event etc.)?** *_c*	**Why did you stop? Please try to remember the exact cause!** *_d*
B428	Sidko plant	1 ❑ yes<br>0 ❑ no				
B429	Pond sandfilter	1 ❑ yes<br>0 ❑ no				
B430	Piped water supply system	1 ❑ yes<br>0 ❑ no				
B431	Dugwell	1 ❑ yes<br>0 ❑ no				
B432	Deep tubewell	1 ❑ yes<br>0 ❑ no				
B433	Community rain-water harvesting system	1 ❑ yes<br>0 ❑ no				

Current water consumption of the family

Interviewer: *Ask the respondent to show you, which vessel he or she uses for drinking.*

B177 Estimated contents of the vessel used for **drinking** *(interviewer estimate):* .... liters.

Interviewer: *Ask the respondent to show you, which vessel he or she uses for pouring water for cooking.*

B178 Estimated contents of the vessel used for **pouring water for cooking** *(interviewer estimate):* .... liters.

Please only fill in the first column of the tables (number of vessels).	*B179*<br>How many vessels of the following water sources do you and others living in your household **drink** per day?			*B180*<br>How many vessels of the following water sources do you and others living in your household **use for cooking** per day?		
	Vessels *1*	Liters *2*	(%) *3*	Vessels *1*	Liters *2*	(%) *3*
a **Total:**			*100*			*100*
b Water from household As filter						
c Water from community As filter (Sidko)						
d Tubewell water from neighbor (green/uncontaminated)						
e Water from untested tubewell						
f Bottled water						
g Sand filtered pond water						
h Rain water (from their own rainwater harvesting system)						
i Deep tubewell water						
j Dugwell water						

Please only fill in the first column of the tables (number of vessels).	*B181*<br>How many vessels of the following water sources do you and others living in your household **drink** per day?			*B182*<br>How many vessels of the following water sources do you and others living in your household **use for cooking** per day?		
	Vessels *1*	Liters *2*	(%) *3*	Vessels *1*	Liters *2*	(%) *3*
k Water from piped water supply system						
l Water from re-sunk, private tubewell						
m Water from pond/river (unfiltered)						
n Tubewell water (red/contaminated)						
o Rain water (from community rainwater harvesting system)						
p Other:.....................................						

For USERS of arsenic mitigation options (= presently taking water from an arsenic mitigation option):

- ***For people who use one option: Please select the appropriate sheets for the option they are using.***
- ***For people who use several options: Please select the appropriate sheets for the option the respondent is mainly using for drinking and cooking and attach them to the questionnaire.***
- ***EXCEPTION: people who use an arsenic mitigation option that is not listed here (p = other): thank respondent, ask if she/he has any questions, answer them and end the interview.***

For NON-USERS of arsenic mitigation options (= people who are presently NOT taking water form an arsenic mitigation option):

- ***If they previously used an option: Please select the appropriate sheets for the option they previously used!***
- ***If they have never used an option: Please ask them, which option they are most likely to use of the options that are available to them (information from B417-B433)!***

For DEEP TUBEWELL USERS that use only deep tubewells for drinking and cooking:

- ***If they previously used any other option (information from B417-B433): Please select the appropriate sheets for the option they previously used!***
- ***If they have never used any other option (information from B417-B433): Thank the respondent, ask if he or she has any questions, answer them and end the interview!***

Household Filter Questionnaire *(Sono/ Alcan/ Read-F)*

Advantages and disadvantages of drinking water from red tubewell / un-tested well

a. What are the advantages and disadvantages of drinking **water from red tubewell / untested well?**

r. Which of them are **the three most important advantages / disadvantages?** ***1 = most important, 2 = 2nd most important, 3 = 3rd most important***

Drinking **water from red tubewell / untested well**	
**Advantages**	**Ranking** *r*
B192	
B193	
B194	
B195	
B196	
**Disadvantages**	**Ranking** *r*
B197	
B198	
B199	
B200	
B201	

Water from red tubewell / untested tubewell

If family drinks water or cooks with water from an untested tubewell:

B204 Why haven't you tested your tubewell for arsenic?

……..

……..

B207 How much did you pay for installation of the red tubewell / untested tubewell?

………………Tk   9 ❑ I don't know   888 ❑ Doesn't own a tubewell.

B208 How much do you pay for maintenance of the red tubewell / untested tubewell per year? ………………Tk

B209 How much do you like or dislike **drinking** water from the red tubewell / untested tubewell?

Rather dislike it			Rather neutral			Rather like it		
-4❑	-3❑	-2❑	-1❑	0❑	1❑	2❑	3❑	4❑
I dislike it very much	I dislike it	I quite dis-like it	I rather dis-like it	I neither dislike it nor do I like it	I rather like it	I quite like it	I like it	I like it very much

B210 How much do you like or dislike the **taste** of water from the red tubewell / untested tubewell?

Rather dislike it			Rather neutral			Rather like it		
-4❑	-3❑	-2❑	-1❑	0❑	1❑	2❑	3❑	4❑
I dislike it very much	I dislike it	I quite dis-like it	I rather dis-like it	I neither dislike it nor do I like it	I rather like it	I quite like it	I like it	I like it very much

B211 How much do you like or dislike the **smell** of water from the red tubewell / untested tubewell?

Rather dislike it			Rather neutral			Rather like it		
-4❑	-3❑	-2❑	-1❑	0❑	1❑	2❑	3❑	4❑
I dislike it very much	I dislike it	I quite dis-like it	I rather dis-like it	I neither dislike it nor do I like it	I rather like it	I quite like it	I like it	I like it very much

B212 How much do you like or dislike the **temperature** of water from the red tubewell / untested well?

Rather dislike it			Rather neutral			Rather like it		
-4❑	-3❑	-2❑	-1❑	0❑	1❑	2❑	3❑	4❑
I completely dislike it	I dislike it	I quite dis-like it	I rather dis-like it	I neither dislike it nor do I like it	I rather like it	I quite like it	I like it	I like it very much

B213 How much do you like or dislike the **color** of water from the red tubewell / untested tubewell?

Rather dislike it			Rather neutral			Rather like it		
-4❑	-3❑	-2❑	-1❑	0❑	1❑	2❑	3❑	4❑
I dislike it very much	I dislike it	I quite dis-like it	I rather dis-like it	I neither dislike it nor do I like it	I rather like it	I quite like it	I like it	I like it very much

B214 How high or low are the chances that you develop an illness when drinking water from a red tubewell / an untested tubewell?

Rather low			Rather average			Rather high		
-4❑	-3❑	-2❑	-1❑	0❑	1❑	2❑	3❑	4❑
Very low	Low	Quite low	Rather low	Average	Rather high	Quite high	High	Very high

B215 Why do you think that you are likely / unlikely (***select according to previous answer!***) to develop an ill-ness when drinking water from the red tubewell / untested tubewell?

....

....

B217 How many people of your atio shojun (= relatives), excluding people of your own household, drink water from a red tubewell / untested tubewell?

0❑	1❑	2❑	3❑	4❑
(Almost) nobody (0%)	Some of them (25%)	Half of them (50%)	Most of them (75%)	(Almost) all of them (100%)

B218 How many people **outside your family** drink water from a red tubewell / untested tubewell?

0❑	1❑	2❑	3❑	4❑
(Almost) nobody (0%)	Some of them (25%)	Half of them (50%)	Most of them (75%)	(Almost) all of them (100%)

B219 Let's suppose you drink water from an untested/ red tubewell. Would people who are important to you rather approve or disapprove of this?

Rather disapprove			Rather neutral			Rather approve		
-4❑	-3❑	-2❑	-1❑	0❑	1❑	2❑	3❑	4❑
Nearly all disapprove	Most disap-prove	Significantly more disap-prove	Rather more disapprove	The same amount disapprove and approve	Rather more approve	Significantly more ap-prove	Most ap-prove	Nearly all approve

B221 How good or bad would you say is it to drink water from a red tubewell / untested tubewell?

Rather bad			Rather neutral			Rather good		
-4❑	-3❑	-2❑	-1❑	0❑	1❑	2❑	3❑	4❑
Very bad	Bad	Quite bad	A little bit bad	Neither good nor bad	A little good	Quite good	Good	Very good

B222 How difficult or easy is it to resist drinking water from the red tubewell / untested tubewell?

Rather difficult			Rather neutral			Rather easy		
-4❑	-3❑	-2❑	-1❑	0❑	1❑	2❑	3❑	4❑
Very diffi-cult	Difficult	Quite dif-ficult	Rather difficult	Neither easy nor difficult	Rather easy	Quite easy	Easy	Very easy

B223 I am confident that I can resist drinking water from the red tubewell / untested tubewell from now on.

Rather disagree			Rather neutral			Rather agree		
-4❑	-3❑	-2❑	-1❑	0❑	1❑	2❑	3❑	4❑
I strongly disagree	I disagree	I quite dis-agree	I rather dis-agree	I neither agree nor disagree	I rather agree	I quite agree	I agree	I strongly agree

B224 How strongly do you intend to always drink water from the red tubewell / untested tubewell?

0 ❑ Not at all 1 ❑ Not 2 ❑ A little 3 ❑ Strongly 4 ❑ Very strongly

B225 How strongly do you intend to always cook with water from the red tubewell / untested tubewell?

0 ❑ Not at all 1 ❑ Not 2 ❑ A little 3 ❑ Strongly 4 ❑ Very strongly

B226 Do you think there is something harmful in the water from red tubewells / untested tubewells?
1 ❑ yes 0 ❑ no 9 I don't know

B227 Do you think there is something harmful in the food you cooked with water from red tubewells / untested tubewells? 1 ❑ yes 0 ❑ no 9 I don't know

	B226 Which of these substances does the **water from your red tubewell / untested tubewell** contain (or the one you use, in case you don't own a tubewell):	*B227* Which of these substances does **food cooked with water** from your red tubewell / untested tubewell (or the one you use, in case you don't own a tubewell) contain:
a Arsenic	1 ❑ yes 0 ❑ no 888 ❑ I don't know	1 ❑ yes 0 ❑ no 888 ❑ I don't know
b Iron	1 ❑ yes 0 ❑ no 888 ❑ I don't know	1 ❑ yes 0 ❑ no 888 ❑ I don't know
o Other:...........................	1 ❑ yes 0 ❑ no 888 ❑ I don't know	1 ❑ yes 0 ❑ no 888 ❑ I don't know
	B228 Do you think **the filter** you got is capable to clean the water from:	
a Arsenic	1 ❑ yes 0 ❑ no 888 ❑ I don't know	
b Iron	1 ❑ yes 0 ❑ no 888 ❑ I don't know	
o Other:...........................	1 ❑ yes 0 ❑ no 888 ❑ I don't know	

B216 What do you think of people who have a filter but drink water directly from a red tubewell or untested well without filtering it?

Rather badly			Rather neutral			Rather well		
-4❑	-3❑	-2❑	-1❑	0❑	1❑	2❑	3❑	4❑
I think very badly of them	I think badly of them	I think quite badly of them	I think rather badly of them	I think neither badly nor well of them	I think rather well of them	I think quite well of them	I think well of them	I think very well of them

Advantages and disadvantages of using the filter and drinking filtered water

a. What are the advantages and disadvantages of drinking **filtered water** ?

r. Which of them are **the three most important advantages / disadvantages?** ***1 = most important, 2 = 2nd most important, 3 = 3rd most important***

Using the filter and drinking **filtered water**	
**Advantages**	**Ranking** *r*
B229	
B230	
B231	
B232	
B233	
**Disadvantages**	**Ranking** *r*
B234	
B235	
B236	
B237	
B238	

Filter knowledge

B239 How much does the filter cost? .... Tk.

B240 Does the filter media need changing from time to time? 1 ❑ yes 0 ❑ no 9 ❑ I don't know

B241 ***If yes:*** How often?

0	1	2	3	4	5	6	9
Daily	Weekly	Every two weeks	Monthly	Every six months	Every year	Once in two years	I don't know

B245 ***If yes:*** How much does the refill media cost? .... Tk. 888 ❑ I don't know

B242 Does the filter media need washing from time to time? 1 ❑ yes 0 ❑ no 9 ❑ I don't know

B243 ***If yes:*** How often?

0	1	2	3	4	5	6	9
Daily	Weekly	Every two weeks	Monthly	Every six months	Every year	Once in two years	I don't know

B244 How do you know, when the filter media needs washing?

....

....

B247 What (would you) do you do / whom do you contact when (if) you experience problems regarding filter repairs?

....

....

Filter use

B248 Which filter do you use?

1❑ Sono 2❑ Alcan 3❑ Read-F

B249 Where did you get your filter from?

1❑ MSUK (Sono manufacturer) 2❑ NGO 3❑ DPHE 4❑ UNICEF

B250 Estimated contents of the vessel/jug used for **filling the filter** *(interviewer estimate):* .... liters.

	**Vessel/ Jugs** *a*	**Liters** *b*	**(%)** *c*
B255 How many vessel do you fill into the filter for ....? (other purpose)			

B256 How much did you pay for the filter? __________Tk.

B258 How many times a day do you fill the filter with water? __________times

B264 Who of your household makes the decision if the water is filtered or not?

B264a	Respondent	1❑ yes	0 no
B264b	Husband	1❑ yes	0 no
B264c	Wife	1❑ yes	0 no
B264d	Daughter	1❑ yes	0 no
B264e	Son	1❑ yes	0 no
B264f	Brother	1❑ yes	0 no
B264g	Sister	1❑ yes	0 no
B264h	Father	1❑ yes	0 no
B264i	Mother	1❑ yes	0 no
B264o	Other: …………………………………..		

B271 How strongly do you intend to always cook with filtered water?

0❑ Not at all 1❑ Not 2❑ A little 3❑ Strongly 4❑ Very strongly

B272 I want to cook with filtered water regularly from now on.

Rather disagree			Rather neutral			Rather agree		
-4❑	-3❑	-2❑	-1❑	0❑	1❑	2❑	3❑	4❑
I strongly disagree	I disagree	I quite disagree	I rather disagree	I neither agree nor disagree	I rather agree	I quite agree	I agree	I strongly agree

B273 How often do you need more water for drinking and cooking than is available from the filter?

0 ❑ (Almost) always 1 ❑ Often 2 ❑ Sometimes 3 ❑ (Almost) never

B274 Are there any seasonal peculiarities in using the filter?

……
……

B275 Filling the filter with water is something I do as a matter of habit.

Rather disagree			Rather neutral			Rather agree		
-4❑	-3❑	-2❑	-1❑	0❑	1❑	2❑	3❑	4❑
I strongly disagree	I disagree	I quite dis-agree	I rather dis-agree	I neither agree nor disagree	I rather agree	I quite agree	I agree	I strongly agree

B276 Do you fill the filter automatically?

0❑	1❑	2❑	3❑	4❑
Not at all automati-cally	Not auto-matically	Quite auto-matically	Automati-cally	Very auto-matically

B277 When do you normally fill the filter with water (e.g. after getting up, after cleaning etc.)?

....

....

B278 How often do you fill the filter at these moments?

0 ❑ (Almost) never 1 ❑ Often 2 ❑ Sometimes 3 ❑ (Almost) always

B279 How much do other habits hinder you to fill your filter with water?

0 ❑ Not at all 1 ❑ A little 2 ❑ Medium 3 ❑ Much 4 ❑ Very much

B280 How difficult is it to remember filling the filter with water?

0 ❑ Very difficult 1 ❑ Difficult 2 ❑ Medium 3 ❑ Not so difficult 4 ❑ Not difficult at all

B281 How often does it happen that you forget to fill the filter with water?

0 ❑ (Almost) always 1 ❑ Often 2 ❑ Sometimes 3 ❑ (Almost) never

B282 How often did you forget to fill the filter with water in the last two weeks? ______________times

B283 Is there something which helps you remind to fill the filter? 1 ❑ yes 0 no

B284 ***If yes:*** what or who reminds you to fill the filter?

____

B285 How important is it for you to fill the filter regularly?

Rather unimportant			Rather neutral			Rather important		
-4❑	-3❑	-2❑	-1❑	0❑	1❑	2❑	3❑	4❑
Totally un-important	Very unim-portant	Quite unim-portant	Unimportant	Neither of both	Important	Quite impor-tant	Very impor-tant	Totally im-portant

B286 Do you feel committed to use the filter?

0 ❑ Not at all 1 ❑ A little 2 ❑ Medium 3 ❑ Much 4 ❑ Very much

B287 How annoyed do you feel if you forget to fill the filter?

0 ❑ Not at all 1 ❑ A little 2 ❑ Medium 3 ❑ Much 4 ❑ Very much

B288 How often does it happen to you that you want to fill the filter but then you prefer to do something else?

0 ❑ (Almost) always 1 ❑ Often 2 ❑ Sometimes 3 ❑ (Almost) never

B289 What is it that you prefer to do in these moments?

____

B293 How strongly do you intend to always have water in the filter?

0 ❑ Not at all 1 ❑ Not 2 ❑ A little 3 ❑ Strongly 4 ❑ Very strongly

B294 I want to fill the filter with water regularly from now on.

Rather disagree			Rather neutral			Rather agree		
-4 ❑	-3 ❑	-2 ❑	-1 ❑	0 ❑	1 ❑	2 ❑	3 ❑	4 ❑
I strongly disagree	I disagree	I quite dis-agree	I rather dis-agree	I neither agree nor disagree	I rather agree	I quite agree	I agree	I strongly agree

Various barriers make it hard to use the filter. How certain are you that you can use the filter? **Are you sure that…**	
B290 …you can use the filter to prepare as much arsenic free water as you need within the next year?	0 ❑ Very unsure<br>1 ❑ Unsure<br>2 ❑ A little bit unsure<br>3 ❑ Sure<br>4 ❑ Very sure
B291 …you can use the filter to prepare as much arsenic free water as you need within the next month?	0 ❑ Very unsure<br>1 ❑ Unsure<br>2 ❑ A little bit unsure<br>3 ❑ Sure<br>4 ❑ Very sure
B292 …you can use the filter to prepare as much arsenic free water as you need within the next week?	0 ❑ Very unsure<br>1 ❑ Unsure<br>2 ❑ A little bit unsure<br>3 ❑ Sure<br>4 ❑ Very sure
For people who haven't started using the filter:<br><br>**If you intend to start filtering your water in the future, do you have a detailed plan…**	
B295 … **when** during the day to start using the filter?	0 ❑ No detailed plan at all<br>1 ❑ No detailed plan<br>2 ❑ Quite detailed plan<br>3 ❑ Detailed plan<br>4 ❑ Very detailed plan
B296 … **from when on** to start using the filter?	0 ❑ No detailed plan at all<br>1 ❑ No detailed plan<br>2 ❑ Quite detailed plan<br>3 ❑ Detailed plan<br>4 ❑ Very detailed plan
B297 … **how** to start using the filter?	0 ❑ No detailed plan at all<br>1 ❑ No detailed plan<br>2 ❑ Quite detailed plan<br>3 ❑ Detailed plan<br>4 ❑ Very detailed plan

**Have you thought about <u>possible barriers</u> that might interfere with your goal to continuously filter your water? Have you made a detailed plan regarding…**	
B298 … what to do when the filter gets broken?	0 ❑ No detailed plan at all<br>1 ❑ No detailed plan<br>2 ❑ Quite detailed plan<br>3 ❑ Detailed plan<br>4 ❑ Very detailed plan
B299 … how to avoid forgetting to fill the filter?	0 ❑ No detailed plan at all<br>1 ❑ No detailed plan<br>2 ❑ Quite detailed plan<br>3 ❑ Detailed plan<br>4 ❑ Very detailed plan
B300 … what to do when the filter gets clogged?	0 ❑ No detailed plan at all<br>1 ❑ No detailed plan<br>2 ❑ Quite detailed plan<br>3 ❑ Detailed plan<br>4 ❑ Very detailed plan

B301 What do you do to ensure the filter works properly?

……

……

B302 Do you wash the filter media? 1 ❑ yes 0 no

B303 ***If yes:*** Every how many weeks do you wash the filter media? Every…………………weeks

B304 Which parts of the filter have you ever repaired? How much did you pay for the repairs?	***Filter parts***	***Cost***<br>*a*
	_1	**Tk**<br>888 I don't know
	_2	**Tk**<br>888 I don't know
	_3	**Tk**<br>888 I don't know
	9❑ I have not repaired any parts yet	

B305 In your opinion, is repairing the filter expensive or cheap?

Rather expensive			Rather neutral			Rather cheap		
-4❑	-3❑	-2❑	-1❑	0❑	1❑	2❑	3❑	4❑
Very expensive	Expensive	Quite expensive	Rather expensive	Neutral	Rather cheap	Quite cheap	Cheap	Very cheap

B306 How many times was the filter broken in the last year and you couldn't use it? ________times

B307 How many times did you repair the filter in the last year? _________times

B308 How long were you not able to use the filter because it was broken? ....(days)

B309 Why did it take a long time to make the repair(s)?

……

……

B310 If you did not repair the filter: What are the reasons why you didn't repair the filter when it was broken?

……

……

Filter Attitudes

B312 Do you think that using the filter is time-consuming?

0❑	1❑	2❑	3❑	4❑
Not at all time-consuming	Not time-consuming	A little time-consuming	Time-consuming	Very time-consuming

B313 Do you think that using the filter is effortful?

0❑	1❑	2❑	3❑	4❑
Not at all effortful	Not effortful	A little effortful	Effortful	Very effortful

B314 How much do you like or dislike using the filter?

Rather dislike it			Rather neutral			Rather like it		
-4❑	-3❑	-2❑	-1❑	0❑	1❑	2❑	3❑	4❑
I dislike it very much	I dislike it	I quite dis-like it	I rather dis-like it	I neither dislike it nor do I like it	I rather like it	I quite like it	I like it	I like it very much

B315 Considering all the benefits and efforts related to filtering the water, how much do you think is it worthwhile to filter your water?

Rather more effort than benefit			Rather neutral			Rather higher benefit than effort		
-4❑	-3❑	-2❑	-1❑	0❑	1❑	2❑	3❑	4❑
It costs much more effort than benefit	It costs more effort than benefit	It costs quite more effort than benefit	It costs slightly more effort than benefit	The effort and the benefit are about the same	The benefit is slightly higher than the effort	The benefit is quite higher than the effort	The benefit is higher than the effort	The benefit is very much higher than the effort

B316 Does the filter remove all harmful substances (e.g. iron, arsenic)?

0❑	1❑	2❑	3❑	4❑	9❑
No sub-stances are removed	Some sub-stances are removed	Quite a few substances are removed	Many sub-stances are removed	All sub-stances are removed	I don't know

B317 How much do you like or dislike the appearance of the filter?

Rather dislike it			Rather neutral			Rather like it		
-4❑	-3❑	-2❑	-1❑	0❑	1❑	2❑	3❑	4❑
I dislike it very much	I dislike it	I quite dis-like it	I rather dis-like it	I neither dislike it nor do I like it	I rather like it	I quite like it	I like it	I like it very much

B318 What exactly do you like/dislike about the appearance of the filter?

……

……

B319 Is there anything that makes it easier for you to use the filter?

....

....

B320 What do you think of people who use filters to prepare their drinking water?

Rather badly			Rather neutral			Rather well		
-4 ❑	-3 ❑	-2 ❑	-1 ❑	0 ❑	1 ❑	2 ❑	3 ❑	4 ❑
I think very badly of them	I think badly of them	I think quite badly of them	I think rather badly of them	I think neither badly nor well of them	I think rather well of them	I think quite well of them	I think well of them	I think very well of them

B321 How often do you do what other members of your atio shojun (= relatives) except your household members do?

0 ❑ (Almost) never 1 ❑ Sometimes 2 ❑ Often 3 ❑ (Almost) always

B322 How often do you do what other people in your village do?

0 ❑ (Almost) never 1 ❑ Sometimes 2 ❑ Often 3 ❑ (Almost) always

B323 Let's suppose you drink filtered water. Would people who are important to you rather approve or disapprove of this?

Rather disapprove			Rather neutral			Rather approve		
-4 ❑	-3 ❑	-2 ❑	-1 ❑	0 ❑	1 ❑	2 ❑	3 ❑	4 ❑
Nearly all disapprove	Most disapprove	Significantly more disapprove	Rather more disapprove	The same amount disapprove and approve	Rather more approve	Significantly more approve	Most approve	Nearly all approve

B325 Are there specific persons that are important for you, who would disapprove the use of the filters?

1 ❑ yes 0 no

B326 ***If yes:*** Who?____

B403 Are there specific persons that are important for you, who would approve of you using the filter?

1 ❑ yes 0 no

B404 If yes: Who? ____

B327 It is expected of me that I use the filter.

Rather disagree			Rather neutral			Rather agree		
-4 ❑	-3 ❑	-2 ❑	-1 ❑	0 ❑	1 ❑	2 ❑	3 ❑	4 ❑
I strongly disagree	I disagree	I quite disagree	I rather disagree	I neither agree nor disagree	I rather agree	I quite agree	I agree	I strongly agree

B328 To what extent do you see yourself as being capable of using the filter to prepare as much water as you need?

0 ❑ Not at all capable 1 ❑ Not capable 2 ❑ Quite capable 3 ❑ Capable 4 ❑ Very capable

B329 How difficult or easy is it to use the filter?

Rather difficult			Rather neutral			Rather easy		
-4 ❑	-3 ❑	-2 ❑	-1 ❑	0 ❑	1 ❑	2 ❑	3 ❑	4 ❑
Very difficult	Difficult	Quite difficult	Rather difficult	Neither easy nor difficult	Rather easy	Quite easy	Easy	Very easy

Attitudes drinking filtered water

B330 How much do you like or dislike drinking filtered water?

Rather dislike it			Rather neutral			Rather like it		
-4 ❑	-3 ❑	-2 ❑	-1 ❑	0 ❑	1 ❑	2 ❑	3 ❑	4 ❑
I dislike it very much	I dislike it	I quite dis-like it	I rather dis-like it	I neither dislike it nor do I like it	I rather like it	I quite like it	I like it	I like it very much

B331 How much do you like or dislike the **taste** of filtered water?

Rather dislike it			Rather neutral			Rather like it		
-4 ❑	-3 ❑	-2 ❑	-1 ❑	0 ❑	1 ❑	2 ❑	3 ❑	4 ❑
I dislike it very much	I dislike it	I quite dis-like it	I rather dis-like it	I neither dislike it nor do I like it	I rather like it	I quite like it	I like it	I like it very much

B332 How much do you like or dislike the **smell** of filtered water?

Rather dislike it			Rather neutral			Rather like it		
-4 ❑	-3 ❑	-2 ❑	-1 ❑	0 ❑	1 ❑	2 ❑	3 ❑	4 ❑
I dislike it very much	I dislike it	I quite dis-like it	I rather dis-like it	I neither dislike it nor do I like it	I rather like it	I quite like it	I like it	I like it very much

B333 How much do you like or dislike the **temperature** of filtered water?

Rather dislike it			Rather neutral			Rather like it		
-4 ❑	-3 ❑	-2 ❑	-1 ❑	0 ❑	1 ❑	2 ❑	3 ❑	4 ❑
I dislike it very much	I dislike it	I quite dis-like it	I rather dis-like it	I neither dislike it nor do I like it	I rather like it	I quite like it	I like it	I like it very much

B334 How much do you like or dislike the **color** of filtered water?

Rather dislike it			Rather neutral			Rather like it		
-4 ❑	-3 ❑	-2 ❑	-1 ❑	0 ❑	1 ❑	2 ❑	3 ❑	4 ❑
I dislike it very much	I dislike it	I quite dis-like it	I rather dis-like it	I neither dislike it nor do I like it	I rather like it	I quite like it	I like it	I like it very much

B335 How high or low are the chances that you develop an illness when drinking filtered water?

Rather low			Rather average			Rather high		
-4 ❑	-3 ❑	-2 ❑	-1 ❑	0 ❑	1 ❑	2 ❑	3 ❑	4 ❑
Very low	Low	Quite low	Rather low	Average	Rather high	Quite high	High	Very high

B336 Why do you think that you are likely / unlikely (***select according to previous answer!***) to develop an ill-ness when drinking filtered water?

....

....

B337 Do you have any reservations about drinking filtered water? 1 ❑ yes 0 no

B338 ***If any reservations:*** What are your reservations?

....

....

B339 How much do you see a need for drinking filtered water?

0 ❑ No need at all 1 ❑ A little need 2 ❑ Quite some need 3 ❑ Much need 4 ❑ Very much need

B340 How many people of your atio shojun (= relatives), excluding people of your household, drink filtered water?

0❑	1❑	2❑	3❑	4❑
(Almost) nobody (0%)	Some of them (25%)	Half of them (50%)	Most of them (75%)	(Almost) all of them (100%)

B341 How many people outside your family drink filtered water?

0❑	1❑	2❑	3❑	4❑
(Almost) nobody (0%)	Some of them (25%)	Half of them (50%)	Most of them (75%)	(Almost) all of them (100%)

B342 Do you think that, over all, people who are important to you rather approve or disapprove that/if you drink filtered water?

Rather disapprove			Rather neutral			Rather approve		
-4❑	-3❑	-2❑	-1❑	0❑	1❑	2❑	3❑	4❑
Nearly all disapprove	Most disap-prove	Significantly more disap-prove	Rather more disapprove	The same amount disapprove and approve	Rather more approve	Significantly more ap-prove	Most ap-prove	Nearly all approve

---

B346 How important is it for you to drink filtered water regularly?

0 ❑ Not important at all 1 ❑ Not very important 2 ❑ Quite important 3 ❑ Important 4 ❑ Very important

B347 How important is it for you to present filtered water to your guests?

0 ❑ Not important at all 1 ❑ Not very important 2 ❑ Quite important 3 ❑ Important 4 ❑ Very important

B348 How proud or ashamed are you to offer filtered water to your guests?

Rather ashamed			Rather neutral			Rather proud		
-4❑	-3❑	-2❑	-1❑	0❑	1❑	2❑	3❑	4❑
Very ashamed	Ashamed	Quite ashamed	Rather ashamed	Neither ashamed nor proud	Rather proud	Quite proud	Proud	Very proud

B349 What would your guests think of you if you did not have filtered water?

Rather badly			Rather neutral			Rather well		
-4❑	-3❑	-2❑	-1❑	0❑	1❑	2❑	3❑	4❑
They would think very badly of me	They would think badly of me	They would think quite badly of me	They would think a little badly of me	They would think neither well nor badly of me	They would think a little well of me	They would think quite well of me	They would think well of me	They would think very well of me

B350 How good or bad would you say is it to drink filtered water?

Rather bad			Rather neutral			Rather good		
-4❑	-3❑	-2❑	-1❑	0❑	1❑	2❑	3❑	4❑
Very bad	Bad	Quite bad	A little bit bad	Neither good nor bad	A little good	Quite good	Good	Very good

B351 How certain are you that drinking only filtered water prevents you from getting arsenicosis?

0 ❑ Not at all certain 1 ❑ Not very certain 2 ❑ Quite certain 3 ❑ Certain 4 ❑ Very certain

B352 How strongly do you intend to always drink filtered water?

0 ❑ Not at all 1 ❑ Not 2 ❑ A little 3 ❑ Strongly 4 ❑ Very strongly

B353 I want to drink filtered water regularly from now on.

Rather disagree			Rather neutral			Rather agree		
-4 ❑	-3 ❑	-2 ❑	-1 ❑	0 ❑	1 ❑	2 ❑	3 ❑	4 ❑
I strongly disagree	I disagree	I quite dis-agree	I rather dis-agree	I neither agree nor disagree	I rather agree	I quite agree	I agree	I strongly agree

Willingness to pay

B354 What would be the maximum amount you would be willing to pay for a new filter? ______________Tk

B355 Which price would you find reasonable to pay for a new filter? ________________Tk

B356 Do you think 700 Taka for a new filter which cleans your drinking water is too cheap, too expensive or just about right?

Rather cheap			About right			Rather expensive		
-4 ❑	-3 ❑	-2 ❑	-1 ❑	0 ❑	1 ❑	2 ❑	3 ❑	4 ❑
Much too cheap	Cheap	Quite cheap	Rather cheap	Just right	Rather expensive	Quite expensive	Expensive	Too expensive

B357 What would be the maximum amount you would be willing to pay for a refill of the filter media?
_______________Tk 888 ❑ I don't know

B358 Which price would you find reasonable to pay for a refill of the filter media? ________________Tk
888 ❑ I don't know

B359 What do you think about the price of 700 Taka for a refill of the filter media?

0 ❑ Very low price 1 ❑ Low price 2 ❑ A modest price 3 ❑ High price 4 ❑ Very high price

B360 What would be the maximum amount you would be willing to pay for an arsenic test of your water source?
___________Tk

B361 Which price would you find reasonable to pay for an arsenic test of your water source? ____________Tk

B362 What do you think about the price of 100 Taka for an arsenic test of your water source?

0 ❑ Very low price 1 ❑ Low price 2 ❑ A modest price 3 ❑ High price 4 ❑ Very high price

B363 What would be the maximum amount you would be willing to pay per year for exchanging broken parts of the filter? _____________Tk.

B364 And now, after all we discussed, would you now be willing to pay ________________(insert max. amount from items above) for safe drinking water?

0 ❑ Certainly not 1 ❑ Probably not 2 ❑ Probably yes 3 ❑ Certainly yes

B367 Who of your household makes financial decisions?

0 ❑ male head of household 1 ❑ female head of household 2 ❑ other male 3 ❑ other female

B368 How much do you think NGOs should support you?

0 ❑ Not at all 1 ❑ A little 2 ❑ Medium 3 ❑ Much 4 ❑ Very much

B369 Who do you think is responsible for improving the water quality of your drinking water?

B370 Who do you think is responsible for changing the filter media?

B371 Who do you think is responsible for repairing the filter?

B372 What should be done in your opinion so that people who have a filter really use it?

………

………

………

Campaigns

B373 Were you ever visited by people from NGOs or the government who talked to you about water-related issues? Please tell me, when you were visited, who visited you, and what the visits were about (what the people told you, whether they installed anything or gave you any materials etc.).

	**When did they visit?** (Date) *a*	**Who visited?** *b*	**What was the visit about?** *c*	**What was installed / given?** *d*
_1				
_2				
_3				
_4				

B434 What kind of **community or group activities** regarding water-related issues were or have been going on in your area in the past 5 years (*e.g. focus group discussion, arsenic mitigation committee, etc.)*? Who initiated these activities? Please describe!

………

………

………

Health status and awareness

B374 How often do you talk positively about water treatment and health with others?

0 never
1 less often than every month
2 every month
3 every 3 weeks
4 every 2 weeks
5 every week
6 every 1 to 3 days

B375 Can you tell me what arsenic is?

____

____

B376 What are the effects of arsenic on your body?

____

____

B377 What do you think causes these effects of arsenic?

____

____

B378 How long does it take until arsenic has negative effects on the body?

____

____

B379 What can be done to avoid arsenic and its harmful effects?

____

____

Explain to the respondent about arsenic, its health-effects and how it can be avoided!

B380 What do you think of people who have arsenicosis?

Rather badly			Rather neutral			Rather well		
-4 ❑	-3 ❑	-2 ❑	-1 ❑	0 ❑	1 ❑	2 ❑	3 ❑	4 ❑
I think very badly of them	I think badly of them	I think quite badly of them	I think rather badly of them	I think neither badly nor well of them	I think rather well of them	I think quite well of them	I think well of them	I think very well of them

B381 Do you have arsenicosis? 1 ❑ yes 0 ❑ no 9 ❑ I don't know

B382 How many people of your household have arsenicosis? ___________people 888 ❑ I don't know

B383 How many people outside your family have arsenicosis? ___________people 888 ❑ I don't know

Disadvantages of arsenicosis	
1. In your opinion what are the disadvantages of having arsenicosis?<br>2. Which one is the most important (=1), the 2nd most important (=2) and the 3rd most important disadvantage (=3)?	**Ranking**<br>*r*
B384	
B385	
B386	
B387	

Imagine that you contracted arsenicosis, how severe would be the impact on…

B388 … your life in general?

0 ❑ Not severe at all   1 ❑ Not severe   2 ❑ Quite severe   3 ❑ Severe   4 ❑ Very severe

B389 … your social life?

0 ❑ Not severe at all   1 ❑ Not severe   2 ❑ Quite severe   3 ❑ Severe   4 ❑ Very severe

B390 … your economic situation?

0 ❑ Not severe at all   1 ❑ Not severe   2 ❑ Quite severe   3 ❑ Severe   4 ❑ Very severe

B392 How high or low do you feel are the chances that you get arsenicosis?

Rather low			Rather average			Rather high		
-4❑	-3❑	-2❑	-1❑	0❑	1❑	2❑	3❑	4❑
Very low	Low	Quite low	Rather low	Average	Rather high	Quite high	High	Very high

B365 Why do you think that the chances that you develop arsenicosis are high/low?

....

....

B393 How high or low are the chances that someone of your family develops arsenicosis?

Rather low			Rather average			Rather high		
-4❑	-3❑	-2❑	-1❑	0❑	1❑	2❑	3❑	4❑
Very low	Low	Quite low	Rather low	Average	Rather high	Quite high	High	Very high

B394 If you could avoid having arsenicosis, how much would you be willing to pay for it? _______________Tk

B395 If you could avoid having arsenicosis, how much effort would you be willing to give?

0 ❑ No effort   1 ❑ A little effort   2 ❑ Medium effort   3 ❑ Much effort   4 ❑ Very much effort

Preferences of mitigation options

a. Which water options do you know that provide arsenic-free drinking water? ***<u>Don't</u> read this list!***

b. Which are your most, 2nd most and 3rd most preferred arsenic-free water options? ***1 = the most preferred, 2 = 2nd most preferred, 3 = 3rd most preferred, 0 = option was not mentioned***

c. What are the advantages of these options?

r. Which advantages are most, 2nd most and 3rd most important for you? ***1 = most important, 2 = 2nd most important, 3 = 3rd most important***

d. What are the disadvantages of these options?

r. Which disadvantages are most, 2nd most and 3rd most important for you? ***1 = most important, 2 = 2nd most important, 3 = 3rd most important***

**Water option mentioned?**	**Pref. ranking** *b*	**Advantages** *c*	*Import. ranking _r*	**Disadvantages** *d*	*Import. ranking _r*
B181 Household arsenic re-moval filter [1] ❑ yes [0] ❑ no		*1*_____<br>*2*_____<br>*3*_____<br>*4*_____	___<br>___<br>___<br>___	*1*_____<br>*2*_____<br>*3*_____<br>*4*_____	___<br>___<br>___<br>___
B182 Community arsenic removal filter (Sidko) [1] ❑ yes [0] ❑ no		*1*_____<br>*2*_____<br>*3*_____<br>*4*_____	___<br>___<br>___<br>___	*1*_____<br>*2*_____<br>*3*_____<br>*4*_____	___<br>___<br>___<br>___
B183 Green/uncontaminated tubewell from neighbor [1] ❑ yes [0] ❑ no		*1*_____<br>*2*_____<br>*3*_____<br>*4*_____	___<br>___<br>___<br>___	*1*_____<br>*2*_____<br>*3*_____<br>*4*_____	___<br>___<br>___<br>___

Water option mentioned?	Pref. ranking<br>*b*	Advantages<br>*c*	*Import. ranking*<br>*_r*	Disadvantages<br>*d*	*Import. ranking*<br>*_r*
B184 Bottled water [1] ❑ yes [0] ❑ no		*1*___<br>*2*___<br>*3*___<br>*4*___	___<br>___<br>___<br>___	*1*___<br>*2*___<br>*3*___<br>*4*___	___<br>___<br>___<br>___
B185 Pond Sand Filter [1] ❑ yes [0] ❑ no		*1*___<br>*2*___<br>*3*___<br>*4*___	___<br>___<br>___<br>___	*1*___<br>*2*___<br>*3*___<br>*4*___	___<br>___<br>___<br>___
B186 Household rainwater harvesting system [1] ❑ yes [0] ❑ no		*1*___<br>*2*___<br>*3*___<br>*4*___	___<br>___<br>___<br>___	*1*___<br>*2*___<br>*3*___<br>*4*___	___<br>___<br>___<br>___
B187 Deep tubewell [1] ❑ yes [0] ❑ no		*1*___<br>*2*___<br>*3*___<br>*4*___	___<br>___<br>___<br>___	*1*___<br>*2*___<br>*3*___<br>*4*___	___<br>___<br>___<br>___

**Water option mentioned?**	**Pref. ranking** *b*	**Advantages** *c*	*Import. ranking _r*	**Disadvantages** *d*	*Import. ranking _r*
B188 Dugwell [1] ❑ yes [0] ❑ no		*1*______<br>*2*______<br>*3*______<br>*4*______	____<br>____<br>____<br>____	*1*______<br>*2*______<br>*3*______<br>*4*______	____<br>____<br>____<br>____
B189 Piped water supply [1] ❑ yes [0] ❑ no		*1*______<br>*2*______<br>*3*______<br>*4*______	____<br>____<br>____<br>____	*1*______<br>*2*______<br>*3*______<br>*4*______	____<br>____<br>____<br>____
B190 Re-sunk, arsenic-free tubewell [1] ❑ yes [0] ❑ no		*1*______<br>*2*______<br>*3*______<br>*4*______	____<br>____<br>____<br>____	*1*______<br>*2*______<br>*3*______<br>*4*______	____<br>____<br>____<br>____
B191 Other:____________		*1*______<br>*2*______<br>*3*______<br>*4*______	____<br>____<br>____<br>____	*1*______<br>*2*______<br>*3*______<br>*4*______	____<br>____<br>____<br>____

Data of the household

B121 Coordinates: ***Mark waypoint on GPS!***

B122 Address (or description): ....

....

B123 Number of persons living in the household (incl. children) (TOTAL): ....

B124 How many rooms does your household have? ....

B125 Monthly expenditure:............... Tk 9 ❑ I don't know

B126 Monthly income: ....Tk 9 ❑ I don't know

How many of the following commodities do you or any member of your household own?

B127.................... House: ....

B128.................... Bicycle: ....

B129.................... Television (B/W): ....

B130.................... Television (color): ....

B131.................... Refrigerator:...

B132.................... Motorcycle: ....

B133.................... Sewing machine: ....

B134.................... Mobile phone:...

B135.................... Wardrobe:...

B136.................... Tables:..

B137.................... Chairs: ....

B138.................... Watches/clocks: ....

B139.................... Beds: ....

B140.................... Sleeping cots:...

B141 Do you have electricity? 1 ❑ yes 0 ❑ no

B142 Type of house floor (main): 1 ❑ Earthen 2 ❑ Bamboo 3 ❑ Wood 4 ❑ Cement 5 ❑ Bricks 6 ❑ Other:..

B143 Type of house wall: 1 ❑ Jute sticks/cane/bamboo 2 ❑ Earth 3 ❑ Wood 4 ❑ Tin 5 ❑ Brick/cement/block/concrete 6 ❑ Other:..

B144 Type of house ceiling: 1 ❑ Jute sticks/bamboo/polyethylene 2 ❑ Tin 3 ❑ Cement/brick 4 ❑ Other:..

B145 What fuel do you cook with? 1 ❑ Wood 2 ❑ Crop residue/grass/dung 3 ❑ Liquid gas/gas

B146 How much land does your household own (incl. homestead land): ………………………..decimals

Comments & recommendations

B396 Do you have any comments for us?

....

....

....

B265 ***Observation:*** Is the filter filled?

2 yes, it was filled a while ago 1 it was filled just now 0 no, filter is dry

B311 Is the filter operational? Any broken parts? (observed)

0 ❑ No, the filter is totally broken and out of order

1 ❑ Yes, the filter is working but some parts are broken

2 ❑ Yes, the filter is working, nothing is broken

B398 Number of people present during interview: ……………………… End time: ....

Official use:			
**Checked:** ❑ yes	Initials:	**Data entered:** ❑ yes	Initials: ....
