# Supplementary material for: Predicting water consumption habits for seven arsenic-safe water options in Bangladesh
Source: BMC Public Health. 2013 May 1;13:417. doi: 10.1186/1471-2458-13-417 (PMC3649919; doi:10.1186/1471-2458-13-417)
Supplement: Additional file 2 — Supporting information. The file contains a table with detailed definitions of all psychological constructs investigated, including their operationalization and internal consistency measures. Furthermore, the parameter estimates and their confidence intervals from all regression models are displayed and discussed. [file 1471-2458-13-417-S2.docx]

SUPPORTING INFORMATION

**Predicting Water Consumption Habits for Seven Arsenic-Safe Water Options in Bangladesh**

Jennifer Inauen^1,2,*^, Robert Tobias^1^, & Hans-Joachim Mosler^1^

^1^ Environmental and Health Psychology, Department of Environmental Social Sciences, Eawag: Swiss Federal Institute of Aquatic Science & Technology, Überlandstrasse 133, Dübendorf 8600, Switzerland

^2^ Developmental and Health Psychology, Department of Psychology, University of Konstanz, P.O. Box 5560, Konstanz 78457, Germany

^*^ Corresponding author. Developmental and Health Psychology, Department of Psychology, University of Konstanz, P.O. Box 5560, Konstanz 78457, Germany, jennifer.inauen@uni-konstanz.de

Number of text pages: 2

Number of figures: 0

Number of tables: 2

Total number of pages SI: 6

**Table S-1. Definition and Operationalization of Constructs**

| **Construct** |  | **Definition** |  | **Items** |  | **Answering options** | **Cronbach's Alpha** |
| --- | --- | --- | --- | --- | --- | --- | --- |
|  |  |  |  |  |  |  |  |
| Habitual behavior |  | A continuum expressing the combination of a person's current arsenic-safe water consumption, as well as its automaticity and regularity. It reaches from not performing a behavior with zero habit strength (i.e. no prior experience of performing it) to an always performed behavior with great habit strength. |  | Averaged sum scale of current arsenic-safe water consumption, perceived habit, automaticity and regularity. |  | 0 = not currently consuming arsenic-safe water and no habit to 4 = currently consuming arsenic-safe water with maximum habit strength | 0.908 |
|  |  |  |  | Current arsenic-safe water consumption: |  |  |  |
|  |  |  |  | 1. How many vessels of drinking water do you collect from … (arsenic-contaminated shallow tubewell; arsenic-safe shallow tubewell; deep tubewell; pond sand filter; dug well; household arsenic removal filter; community arsenic removal filter; piped water supply; pond; river; other; in total)? |  | Number of vessels |  |
|  |  |  |  | 2. Calculate proportion of arsenic-safe drinking water of total drinking water consumption. |  | 0 = drinks no arsenic-safe water (0%) to 4 = drinks only arsenic-safe water (100%) |  |
|  |  |  |  | Perceived habit: Filling the filter with water / collecting water from the arsenic-safe water option is something I do as a matter of habit. |  | 0 = I strongly disagree to 4 = I strongly agree |  |
|  |  |  |  | Automaticity: Do you go to collect water from arsenic-safe option automatically? |  | 0 = not at all automatically to 4 = very automatically |  |
|  |  |  |  | Regularity: (Preceding open question: When do you usually go to collect water from the arsenic-safe water option?). How often do you go to collect water from the arsenic-safe option at these moments? |  | 0 = almost never to 4 = almost always |  |
| Severity |  | Perception concerning the seriousness of the consequences of contracting an illness (Brewer et al., 2007; Floyd et al., 2000). |  | Imagine that you contracted arsenicosis, how severe would be the impact on your life in general? |  | 0 = not at all severe to 4 = very severe | 0.951 |
|  |  |  |  | Imagine that you contracted arsenicosis, how severe would be the impact on your social life? |  | 0 = not at all severe to 4 = very severe |  |
|  |  |  |  | Imagine that you contracted arsenicosis, how severe would be the impact on your economic situation? |  | 0 = not at all severe to 4 = very severe |  |
| Vulnerability |  | Perception of personal risk of contracting a particular illness (Brewer et al., 2007; Floyd et al., 2000). |  | How high or low do you feel are the chances that you get arsenicosis? |  | - 4 = very low to 4 = very high | 0.958 |
|  |  |  |  | How high or low are the chances that someone of your family develops arsenicosis? |  | - 4 = very low to 4 = very high |  |

**Table S-1. Definition and Operationalization of Constructs (continued)**

| **Construct** |  | **Definition** |  | **Items** |  | **Answering options** | **Cronbach's Alpha** |
| --- | --- | --- | --- | --- | --- | --- | --- |
| Affective attitude arsenic-safe option (contaminated or untested tubewell) |  | Feelings that arise when performing a behavior or thinking about it (Trafimow & Sheeran, 1998). |  | How much do you like or dislike drinking water from the arsenic-safe option (contaminated or untested tubewell)? |  | - 4 = I dislike it very much to 4 = I like it very much | 0.929 (0.888) |
|  |  |  |  | How much do you like or dislike the taste of water from the arsenic-safe option (contaminated or untested tubewell)? |  | - 4 = I dislike it very much to 4 = I like it very much |  |
|  |  |  |  | How much do you like or dislike the smell of water from the arsenic-safe option (contaminated or untested tubewell)? |  | - 4 = I dislike it very much to 4 = I like it very much |  |
|  |  |  |  | How much do you like or dislike the temperature of water from the arsenic-safe option (contaminated or untested tubewell)? |  | - 4 = I dislike it very much to 4 = I like it very much |  |
|  |  |  |  | How much do you like or dislike the color of water from the arsenic-safe option (contaminated or untested tubewell)? |  | - 4 = I dislike it very much to 4 = I like it very much |  |
| Instrumental attitude arsenic-safe option |  | Beliefs about the benefits and costs of a behavior (Trafimow & Sheeran, 1998). |  | Do you think that using the filter / collecting water from the arsenic-safe option is time-consuming? |  | 0 = not at all time-consuming to 4 = very time-consuming | 0.672 |
|  |  |  |  | Do you think that using the filter / collecting water from the arsenic-safe option is effortful? |  | 0 = not at all effortful to 4 = very effortful |  |
|  |  |  |  | What are the advantages of drinking water from arsenic-contaminated or untested tubewell? |  | Number of advantages of arsenic-contaminated wells |  |
|  |  |  |  | What are the disadvantages of drinking water from arsenic-safe option? |  | Number of disadvantages of arsenic-safe water options |  |
| Injunctive norm arsenic-safe option |  | Perceptions about which behaviors are typically approved ore disapproved (Schultz et al., 2007) |  | How good or bad would you say is it to drink water from the arsenic-safe option? |  | - 4 = very bad to 4 = very good | 0.801 |
|  |  |  |  | How proud or ashamed are you to offer water from the arsenic-safe option to your guests? |  | -4 = very ashamed to 4 = very proud |  |
|  |  |  |  | What do you think of people who use arsenic-safe options? |  | -4 = I think very badly of them to 4 = I think very well of them |  |
| Descriptive norm arsenic-safe option (contaminated or untested tubewell) |  | Perceptions about which behaviors are typically performed (Cialdini, 2003) |  | How many people of your relatives, excl. people of your household, drink water from the arsenic-safe option (the contaminated or untested tubewell)? |  | 0 = (almost) nobody to 4 = (almost) all of them | 0.588 (0.666) |
|  |  |  |  | How many people outside your family drink water from the arsenic-safe option (the contaminated or untested tubewell)? |  | 0 = (almost) nobody to 4 = (almost) all of them |  |
| Note. Cronbach's Alphas in parentheses are for the constructs referring to the contaminated tubewell. | | | | |  |  |  |

**Table S-1. Definition and Operationalization of Constructs (continued)**

| **Construct** |  | **Definition** |  | **Items** |  | **Answering options** | **Cronbach's Alpha** |
| --- | --- | --- | --- | --- | --- | --- | --- |
| Self-efficacy arsenic-safe option |  | The belief in one's capabilities to organize and execute the courses of action required to manage prospective situations (Bandura, 1997) |  | Are you sure that you can use the filter to prepare / that you can collect as much arsenic-safe water as you need within the next year? |  | 0 = not at all sure to 4 = very sure | 0.914 |
|  |  |  |  | Are you sure that you can use the filter to prepare / that you can collect as much arsenic-safe water as you need within the next month? |  | 0 = not at all sure to 4 = very sure |  |
|  |  |  |  | Are you sure that you can use the filter to prepare / that you can collect as much arsenic-safe water as you need within the next week? |  | 0 = not at all sure to 4 = very sure |  |
| Coping planning |  | The presumption of possible barriers and the invention of ways to overcoming them (Schwarzer, 2008) |  | Have you made a detailed plan regarding what to do when the arsenic-safe option gets broken? |  | 0 = no detailed plan at all to 4 = very detailed plan | 0.924 |
|  |  |  |  | Have you made a detailed plan regarding how to avoid forgetting to fill the filter / to collect water from the arsenic-safe option? |  | 0 = no detailed plan at all to 4 = very detailed plan |  |
| Note. Cronbach's Alphas in parentheses are for the constructs referring to the contaminated tubewell. | | | | |  |  |  |

# Analysis of the Estimated Parameter Values

In the paper, the generalizability of the model was only tested based on the fitting abilities (i.e., by comparing the R-squares). However, besides the general information of how well other behaviors can be explained, by using a number of different datasets that all refer to different behaviors, it can also be estimated how much the estimated parameter values vary. Thus, similarly to the confidence estimates for sampling effects, we get indicators for the uncertainty when applying the model to other behaviors.

The parameter values and their confidence intervals are shown in Table S-3. The estimates with data of one behavior missing show a very small variability of the parameter estimates. The absolute differences to the reference estimates (Estimate 1) and their confidence intervals are mostly smaller than 0.05. Only the constants (between –0.38 and +0.45) and, for Estimate 2, the descriptive norm arsenic-safe option (–0.12), show absolute deviations larger than 0.1 from the reference estimates. Compared to the uncertainty due to sampling, which in most cases is more than double in size, the error of applying the estimated parameter values to other behaviors seems of little importance. In fact, only the estimates of the instrumental attitude arsenic-safe option and the descriptive norm arsenic-safe option of Estimate 2, have values outside the 95% confidence bounds of the reference estimates. Thus, the differences of the parameter estimates might be explained by random differences of the samples and not by differences in the explained behaviors.

In the worst-case scenario of having estimated the parameters with rather similar data for a forecast of quite different behaviors (Estimates 9 and 10), the parameter estimates still differ little from the reference estimates. In Estimate 9, only the estimates for the constant (+0.45) and the instrumental attitude arsenic-safe option (–0.11), and in Estimate 10, the estimates for the constant (–0.23) and the descriptive norm arsenic-safe option (–0.11), deviate more than ±0.1 from the reference estimates. Besides the parameter estimates for these variables, the estimate for the self-efficacy arsenic-safe option of Estimate 9 and, for Estimate 10, the estimates for the instrumental attitude arsenic-safe option and the self-efficacy arsenic-safe option are outside the 95% confidence interval of the reference parameter estimates. This indicates the necessity for further investigations of psychological differences and similarities of different options for mitigating the problem of arsenic in drinking water.

To conclude, regarding the variability of the parameter estimates, the model generalizes well. The differences of the parameter values can mostly be explained by differences in the samples. Nevertheless, further analyses of the differences of the behaviors might be valuable.

Table S-2: Estimated parameter values (B) and 95% confidence intervals for different sub-samples

|  | **Estimate** | | | | | | | | | |
| --- | --- | --- | --- | --- | --- | --- | --- | --- | --- | --- |
| **Variables in the equation** | **1** | **2** | **3** | **4** | **5** | **6** | **7** | **8** | **9** | **10** |
| **Parameter estimates** | | | | | | | | | | |
| (Constant) | -0.54 | -0.80 | -0.42 | -0.41 | -0.29 | -0.54 | -0.36 | -0.92 | -0.08 | -0.77 |
| Severity | 0.00 | 0.04 | -0.01 | -0.04 | 0.00 | 0.01 | -0.06 | 0.05 | -0.04 | 0.05 |
| Vulnerability | -0.20 | -0.21 | -0.18 | -0.20 | -0.21 | -0.19 | -0.18 | -0.20 | -0.22 | -0.19 |
| Affective attitude arsenic-safe option | 0.00 | 0.01 | 0.00 | 0.02 | 0.01 | 0.02 | 0.00 | -0.03 | 0.04 | 0.01 |
| Instrumental attitude arsenic-safe option | 0.24 | 0.34 | 0.22 | 0.20 | 0.19 | 0.21 | 0.24 | 0.28 | 0.12 | 0.33 |
| Affective attitude contaminated/untested tubewell | -0.04 | -0.05 | -0.06 | -0.03 | -0.03 | -0.04 | -0.03 | -0.03 | -0.03 | -0.07 |
| Injunctive norm arsenic-safe option | 0.08 | 0.04 | 0.06 | 0.09 | 0.09 | 0.05 | 0.09 | 0.12 | 0.11 | 0.02 |
| Descriptive norm arsenic-safe option | 0.34 | 0.22 | 0.37 | 0.35 | 0.34 | 0.35 | 0.37 | 0.33 | 0.35 | 0.22 |
| Descriptive norm contaminated/untested tubewell | -0.02 | -0.02 | -0.05 | -0.01 | -0.02 | -0.02 | -0.02 | -0.03 | 0.00 | -0.04 |
| Self-efficacy arsenic-safe option | 0.42 | 0.49 | 0.42 | 0.41 | 0.36 | 0.44 | 0.41 | 0.41 | 0.32 | 0.50 |
| Coping planning | 0.03 | 0.02 | 0.02 | 0.01 | 0.03 | 0.03 | 0.02 | 0.06 | 0.02 | 0.04 |
| **Upper limit of 95% confidence interval of parameter estimates** | | | | | | | | | | |
| (Constant) | -0.15 | -0.41 | 0.01 | 0.00 | 0.11 | -0.12 | 0.06 | -0.51 | 0.35 | -0.31 |
| Severity | 0.09 | 0.14 | 0.09 | 0.06 | 0.10 | 0.11 | 0.04 | 0.15 | 0.07 | 0.15 |
| Vulnerability | -0.17 | -0.18 | -0.14 | -0.17 | -0.18 | -0.16 | -0.15 | -0.17 | -0.19 | -0.16 |
| Affective attitude arsenic-safe option | 0.05 | 0.06 | 0.05 | 0.08 | 0.06 | 0.07 | 0.05 | 0.02 | 0.11 | 0.06 |
| Instrumental attitude arsenic-safe option | 0.31 | 0.42 | 0.31 | 0.28 | 0.27 | 0.29 | 0.32 | 0.35 | 0.22 | 0.42 |
| Affective attitude contaminated/untested tubewell | -0.01 | -0.02 | -0.02 | 0.00 | 0.00 | 0.00 | 0.01 | 0.01 | 0.01 | -0.03 |
| Injunctive norm arsenic-safe option | 0.15 | 0.12 | 0.14 | 0.17 | 0.17 | 0.13 | 0.17 | 0.20 | 0.20 | 0.11 |
| Descriptive norm arsenic-safe option | 0.40 | 0.29 | 0.45 | 0.42 | 0.41 | 0.42 | 0.43 | 0.39 | 0.42 | 0.31 |
| Descriptive norm contaminated/untested tubewell | 0.06 | 0.06 | 0.03 | 0.08 | 0.06 | 0.07 | 0.07 | 0.06 | 0.09 | 0.05 |
| Self-efficacy arsenic-safe option | 0.49 | 0.56 | 0.49 | 0.48 | 0.44 | 0.51 | 0.48 | 0.48 | 0.41 | 0.58 |
| Coping planning | 0.09 | 0.08 | 0.09 | 0.08 | 0.09 | 0.10 | 0.08 | 0.12 | 0.09 | 0.11 |
| **Lower limit of 95% confidence interval of parameter estimates** | | | | | | | | | | |
| (Constant) | -0.92 | -1.20 | -0.85 | -0.82 | -0.69 | -0.97 | -0.77 | -1.33 | -0.52 | -1.22 |
| Severity | -0.09 | -0.05 | -0.11 | -0.14 | -0.10 | -0.09 | -0.16 | -0.05 | -0.15 | -0.06 |
| Vulnerability | -0.23 | -0.24 | -0.21 | -0.23 | -0.24 | -0.23 | -0.21 | -0.23 | -0.25 | -0.22 |
| Affective attitude arsenic-safe option | -0.05 | -0.04 | -0.05 | -0.04 | -0.04 | -0.03 | -0.06 | -0.08 | -0.02 | -0.05 |
| Instrumental attitude arsenic-safe option | 0.16 | 0.26 | 0.14 | 0.11 | 0.11 | 0.12 | 0.16 | 0.20 | 0.03 | 0.25 |
| Affective attitude contaminated/untested tubewell | -0.07 | -0.08 | -0.09 | -0.07 | -0.07 | -0.07 | -0.07 | -0.06 | -0.07 | -0.10 |
| Injunctive norm arsenic-safe option | 0.00 | -0.04 | -0.03 | 0.01 | 0.00 | -0.03 | 0.01 | 0.04 | 0.02 | -0.06 |
| Descriptive norm arsenic-safe option | 0.27 | 0.15 | 0.30 | 0.28 | 0.27 | 0.28 | 0.30 | 0.26 | 0.27 | 0.14 |
| Descriptive norm contaminated/untested tubewell | -0.10 | -0.09 | -0.14 | -0.09 | -0.10 | -0.10 | -0.10 | -0.11 | -0.09 | -0.12 |
| Self-efficacy arsenic-safe option | 0.36 | 0.43 | 0.35 | 0.34 | 0.29 | 0.37 | 0.34 | 0.34 | 0.24 | 0.43 |
| Coping planning | -0.03 | -0.04 | -0.05 | -0.06 | -0.04 | -0.03 | -0.04 | -0.01 | -0.06 | -0.03 |
| R^2^ | 0.69 | 0.72 | 0.68 | 0.68 | 0.66 | 0.67 | 0.69 | 0.71 | 0.66 | 0.73 |
| n | 867 | 745 | 741 | 742 | 745 | 742 | 745 | 742 | 620 | 619 |

*Note.* Dependent: habitual use of arsenic-safe drinking water options. Samples in the Estimates: Estimate 1 = all; Estimate 2 = all excl. rainwater harvesting; Estimate 3 = all excl. household arsenic removal; Estimate 4 = all excl. community arsenic removal; Estimate 5 = all excl. pond sand filter; Estimate 6 = all excl. piped water supply; Estimate 7 = all excl. dug well; Estimate 8 = all excl. well-switching; Estimate 9 = all excl community arsenic removal and pond sand filter; Estimate 10 = all excl. rainwater harvesting, and household arsenic removal.
